# Three New Quinazoline-Containing Indole Alkaloids From the Marine-Derived Fungus *Aspergillus* sp. HNMF114

**DOI:** 10.3389/fmicb.2021.680879

**Published:** 2021-06-02

**Authors:** Sha-Sha Liu, Li Yang, Fan-Dong Kong, Jia-Hui Zhao, Li Yao, Zhi-Guang Yuchi, Qing-Yun Ma, Qing-Yi Xie, Li-Man Zhou, Meng-Fei Guo, Hao-Fu Dai, You-Xing Zhao, Du-Qiang Luo

**Affiliations:** ^1^College of Life Science, Key Laboratory of Medicinal Chemistry and Molecular Diagnosis of Ministry of Education, Hebei University, Baoding, China; ^2^Haikou Key Laboratory for Research and Utilization of Tropical Natural Products, Institute of Tropical Bioscience and Biotechnology, CATAS, Haikou, China; ^3^Key Laboratory of Chemistry and Engineering of Forest Products, State Ethnic Affairs Commission, Guangxi Key Laboratory of Chemistry and Engineering of Forest Products, Guangxi Collaborative Innovation Center for Chemistry and Engineering of Forest Products, School of Chemistry and Chemical Engineering, Guangxi University for Nationalities, Nanning, China; ^4^Tianjin Key Laboratory for Modern Drug Delivery & High-Efficiency, Collaborative Innovation Center of Chemical Science and Engineering, School of Pharmaceutical Science and Technology, Tianjin University, Tianjin, China; ^5^Hainan Institute for Tropical Agricultural Resources, CATAS, Haikou, China

**Keywords:** marine-derived fungus, *Aspergillus* sp., indole alkaloids, α-glucosidase inhibitory activity, ryanodine receptor

## Abstract

By feeding tryptophan to the marine-derived fungus *Aspergillus* sp. HNMF114 from the bivalve mollusk *Sanguinolaria chinensis*, 3 new quinazoline-containing indole alkaloids, named aspertoryadins H–J (**1**–**3**), along with 16 known ones (**4**–**19**), were obtained. The structures of the new compounds were elucidated by the analysis of spectroscopic data combined with quantum chemical calculations of nuclear magnetic resonance (NMR) chemical shifts and electron capture detector (ECD) spectra. Structurally, compound **3** represents the first example of this type of compound, bearing an amide group at C-3. Compounds **10** and **16** showed potent α-glucosidase inhibitory activity with IC_50_ values of 7.18 and 5.29 μM, and compounds **13** and **14** showed a clear activation effect on the ryanodine receptor from *Spodoptera frugiperda* (sfRyR), which reduced the [Ca^2+^]_*ER*_ by 37.1 and 36.2%, respectively.

## Introduction

While plants and terrestrial microorganisms have been in the limelight of natural product research for several decades, the marine environment is one of the current hotspots for the bio-prospection of new bioactive molecules, and it is considered to be a new reservoir for drug discovery (Montaser and Luesch, 2011). Marine microorganisms are some of the most prolific sources of structurally novel and biologically active compounds (Bugni and Ireland, 2004; Shen et al., 2009; Arasu et al., 2013; Manimegalai et al., 2013; Xiong et al., 2013).

**FIGURE 1:**
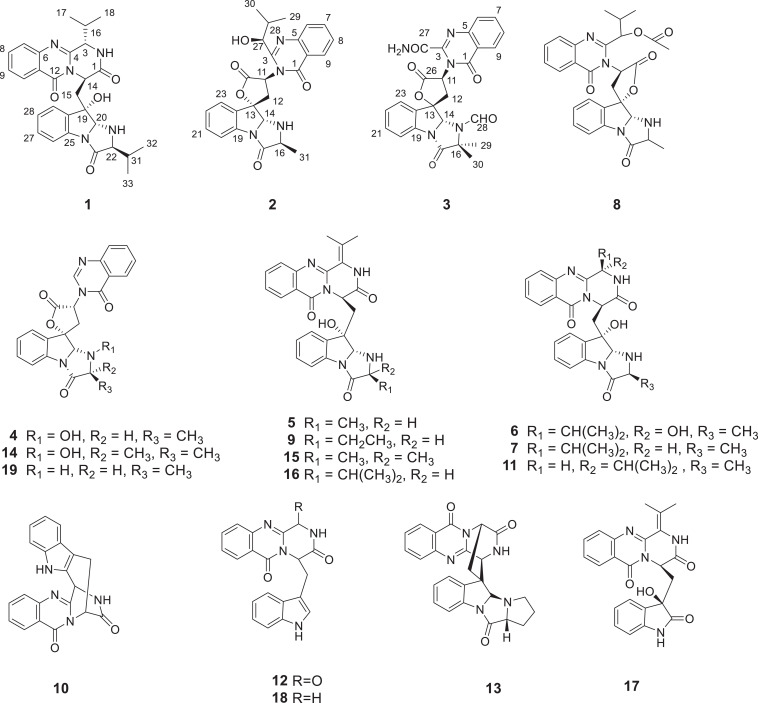
The structures of compounds **1**–**19**.

Among the marine-derived fungi, the genus *Aspergillus* is a prolific source of bioactive secondary metabolites (Blunt et al., 2017). In our search for marine active natural products, the fungus *Aspergillus* sp. HNMF114 was isolated and identified from a bivalve mollusk, *Sanguinolaria chinensis*, collected in Haikou Bay, China. Our previous research (Kong et al., 2019) on the secondary metabolites of this fungus reported a series of quinazoline-containing indole alkaloids. Biogenetically, these compounds are all derived from tryptophan and anthranilic acid, along with other amino acids. It has been reported that feeding tryptophan is beneficial to the production of quinazoline-containing indole alkaloids (Guo et al., 2020). Thus, in order to tap the metabolic potential of this fungus, the continuous chemical investigation on the fermentation broth of the fungus *Aspergillus* sp. HNMF114 supplemented with L-tryptophan was carried out, which led to the isolation of 3 new quinazoline-containing indole alkaloids, aspertoryadins H–J (**1**–**3**), along with 16 known ones, tryptoquivaline H (**4**) (Buttachon et al., 2012), norquinadoline A (**5**) (Peng et al., 2013), *epi*-fiscalin D (**6**) (Qian et al., 2018), *epi*-fiscalin A (**7**) (Buttachon et al., 2012), deoxynortryptoquivaline (**8**) (Lin et al., 2013), scequinadolines D (**9**) (Huang et al., 2017), lapatin A (**10**) (Wu and Ma, 2013), fiscalin A (**11**) (Buttachon et al., 2012), oxoglyantrypine (**12**) (Peng et al., 2013), quinadoline B (**13**) (Koyama et al., 2008), tryptoquivalines L (**14**) (Buttachon et al., 2012), quinadoline A (**15**) (Koyama et al., 2008), scequinadolines E (**16**) (Huang et al., 2017), scequinadoline G (**17**) (Huang et al., 2017), glyantrypine (**18**) (Penn et al., 1992), and tryptoquivalines F (**19**) (Buttachon et al., 2012) (Figure 1). Interestingly, these compounds were all different from those of our previous report (Kong et al., 2019). Herein, the isolation, structure elucidation, and bioactivities of these compounds are reported.

## Materials and Methods

### General Experimental Procedure

Nuclear magnetic resonance (NMR) spectra were obtained on a Bruker AV-600 spectrometer (Bruker, Bremen, Germany) with tetramethylsilane (TMS) as an internal standard. HRESIMS data were determined on a mass spectrometer API QSTAR Pulsar (Bruker, Bremen, Germany). Optical rotations were achieved on a JASCO P-1020 digital polarimeter and IR spectra were recorded on a Shimadzu UV2550 spectrophotometer. UV spectra were measured on a Beckman DU 640 spectrophotometer. Electron capture detector (ECD) data were collected using a JASCO J-715 spectropolarimeter. Silica gel (200–300 mesh, Qingdao Marine Chemical Inc., Qingdao, China) and Sephadex LH-20 (40–70 μm, Merck, Darmstadt, Germany) were used for column chromatography. Semipreparative high-performance liquid chromatography (HPLC) equipped with octadecyl silane column (YMC-pack ODS-A, 10 × 250 mm, 5 μm, 4 mL/min). Spots were visualized by heating silica gel plates sprayed with 10% H_2_SO_4_ in EtOH.

### Fungal Material

The fungal information of *Aspergillus* sp. HNMF114 has been described in our previous report (Kong et al., 2019).

### Fermentation, Extraction, and Isolation

The fungus was cultured was cultured for 30 days in 100 × 1,000 mL erlenmeyer flasks each containing 100 g of rice, 100 mL H_2_O (33 g sea salt, and 2 g tryptophan per liter of tap water) were autoclaved at 121°C for 25 min. The fermented material was extracted three times with EtOAc (20 L for each time) to give 230 g of crude extract. The extract was extracted between petroleum ether and 90% methanol (1:1) to remove the oil. The methanolic extract (119 g) was subjected to a silica gel vacuum-liquid chromatographed (VLC) column, eluting with a stepwise gradient of petroleum ether/EtOAc (10:1, 8:1, 6:1, 4:1, 2:1, 1:1, 1:2, v/v) to afford 10 fractions (Fr.1–Fr.10). Fr.5 (3.0 g) was applied to octadecylsilane (ODS) silica gel with a gradient elution of MeOH-H_2_O (1:4, 1:3, 2:3, 1:1, 3:2, 4:1, 9:1, and 1:0) to get eight subfractions (Fr.5-1-Fr.5-8). Fr. 5-3 (368 mg) was separated by ODS silica gel MeCN-H_2_O (1:3, 2:3, 1:1, 3:2, 4:1, 9:1, and 1:0) to afford two subfractions. Subfraction Fr.5-3-1 was applied to semipreparative HPLC (60% MeOH-H_2_O; 4 mL/min) to give compounds **3** (*t*_*R*_ = 5.8 min; 2.3 mg), **4** (*t*_*R*_ = 7.3 min; 3.3 mg), and **6** (*t*_*R*_ = 8.3 min; 6.1 mg). Fr.5-3-2 was applied to ODS silica gel with gradient elution of MeCN-H_2_O (1:3 to 4:1) semipreparative HPLC (C 60% MeOH-H_2_O; 4 mL/min) to obtain compounds **5** (*t*_*R*_ = 8.2 min; 5.0 mg), **9** (*t*_*R*_ = 10.4 min; 5.3 mg), and **10** (*t*_*R*_ = 10.7 min; 3.4 mg). Fr. 5-5 (56.0 mg) was further chromatographed by silica gel CC eluted with gradient Petroleum ether-EtOAc (10:1, 8:1, 6:1, 4:1, 2:1, 1:1, 1:2, v/v) to afford seven subfractions (Fr.5-5-1–Fr.5-5-7). Fr.5-5-3 were purified by semipreparative HPLC (55% MeOH-H_2_O; 4 mL/min) to yield compounds **11** (*t*_*R*_ = 13.3 min; 1.8 mg), **12** (*t*_*R*_ = 13.8 min; 3.4 mg), **14** (*t*_*R*_ = 15.2 min; 4.7 mg), and **16** (*t*_*R*_ = 17.8 min; 2.7 mg). Fr.6 (2.7 g) was applied to an open ODS column chromatography eluted with stepwise gradient of MeOH-H_2_O (1:5, 1:4, 1:3, 2:3, 1:1, 3:2, 4:1, 9:1, 1:0) to get nine subfractions (Fr.6-1 to Fr.6-9). Fr.6-4 (158 mg) was further separated by Sephadex LH-20 column chromatography, followed by semipreparative HPLC (50% MeCN-H_2_O; containing 0.1% TFA; 4 mL/min) to afford compounds **2** (*t*_*R*_ = 10.3 min; 1.9 mg), **8** (*t*_*R*_ = 17.8 min; 3.8 mg), and **1** (*t*_*R*_ = 27.7 min; 3.4 mg). Fr. 6-5 was subjected to an ODS column chromatography by stepwise gradient elution of MeOH-H_2_O (1:5, 1:4, 1:3, 2:3, 1:1, 3:2, 4:1, 9:1, and 1:0) to get nine subfraction (Fr.6-5-1 to Fr.6-5-9). The subfraction 6-5-4 (53.2 mg) was purified by semipreparative HPLC (45% MeCN-H_2_O; 4 mL/min) to obtain compounds **7** (*t*_*R*_ 8.8 min; 2.3 mg), **13** (*t*_*R*_ 11.4 min; 37.7 mg), **15** (*t*_*R*_ 15.2 min; 4.3 mg), **19** (*t*_*R*_ 15.6 min; 2.8 mg). Compounds **17** (36.2 mg) and **18** (15.3 mg) were combined and crystallized from the Fr.6-5-5 and Fr.6-5-6, respectively.

*Aspertoryadin H (****1****)*: yellow powder solid; [α]25 D -131 (*c* 0.1, MeOH); UV (MeOH) λ_*max*_ (log ε): 200 (3.6), 229 (3.4), 279 (2.9, and 308 (2.6) nm; ECD (MeOH) λ_*max*_ (Δε): 197 (−5.78), 213 (14.45), 232 (−21.54), 330 (−0.17) nm; IR (KBr) υ_*max*_ (cm^–1^): 3,345 (−OH), 2,961 (−CH), 2,925 (−CH), 1,684 (−C=O), 1,602 (−NH), 1,475(−C−N), and 1,031 (−C-C−); ^1^H NMR and^13^C NMR data, see Table 1; HRESIMS *m/z* [M − H]^–^ 500.2310 (calcd for C_2__8_H_30_N_5_O_4_, 500.2303).

**TABLE 1:** ^1^H (600 MHz) and ^13^C NMR (150 MHz) data of compounds **1**–**3**.

	1 (in DMSO-*d*_6_)		2 (in DMSO-*d*_6_)		3 (in DMSO-*d*_6_)	
	δ_*C*_	δ_*H*_ (*J* in Hz)	δ_*C*_	δ_*H*_ (*J* in Hz)	δ_*C*_	δ_*H*_ (*J* in Hz)
1	160.3		161.2		160.9	
3	57.7	4.79 (1H, d, 1.8)	156.6		150.2	
4	151.3					
5			146.2		146.4	
6	146.8		127.3	7.72 (1H, d, 7.8)	128.0	7.82 (1H, d, 9.0)
7	127.2	7.66 (1H, d, 8.4)	135.3	7.89 (1H, t, 7.8)	136.1	7.97 (1H, t, 9.0)
8	134.6	7.83 (1H, dd, 7.2, 8.4)	127.5	7.60 (1H, t, 7.8)	128.9	7.69 (1H, t, 9.0)
9	126.9	7.53 (1H, dd, 7.2, 8.4)	126.2	8.19 (1H, d, 7.8)	126.9	8.27 (1H, d, 9.0)
10	126.3	8.15 (1H, d, 8.4)	120.1		121.4	
11	120.1		54.8	6.07 (1H, t, 9.6)	56.8	5.73 (1H, dd, 10.2, 12.0)
12	160.4		30.8	2.75 (1H, dd, 9.6, 12.6)	27.0	3.31 (1H, dd, 10.2, 16.8)
				3.33 (1H, overlap)		3.40 (1H, dd, 13.2, 16.8)
13			86.1		84.4	
14	52.7	5.35 (1H, dd, 3.6, 7.8)	85.1	5.44 (1H, d, 7.2)	81.5	6.09 (1H, s)
15	37.6	2.80 (1H, dd, 3.6, 14.4)				
		2.67 (1H, dd, 7.8, 14.4)				
16	28.3	2.98 (1H, m)	59.5	3.61 (1H, m)	64.6	
17	19.0	1.16 (3H, d, 7.2)	177.2		172.9	
18	15.4	0.91 (3H, d, 7.2)				
19	74.9		140.9		138.8	
20	83.0	5.01 (1H, d, 7.2)	117.1	7.45 (1H, d, 7.8)	116.8	7.56 (1H, d, 9.0)
21			131.5	7.55 (1H, td, 1.2, 7.8)	132.2	7.61 (1H, t, 9.0)
22	69.9	3.47 (1H, dd, 4.2, 4.2)	126.0	7.38 (1H, td, 1.2, 7.8)	127.2	7.45 (1H, t, 9.0)
23	173.4		125.5	7.67 (1H, d, 7.8)	127.3	8.01 (1H, d, 9.0)
24			132.3		133.9	
25	137.7					
26	114.9	7.36 (1H, d, 7.8)	171.1		171.5	
27	129.6	7.33 (1H, t, 7.8)	80.2	4.35 (1H, dd, 4.8, 9.6)	163.5	
28	124.8	7.13 (1H, t, 7.8)	32.6	2.14 (1H, m)	162.5	8.64 (1H, s)
29	124.9	7.43 (1H, d, 7.8)	19.7	1.14 (3H, d, 6.0)	25.9	1.53 (3H, s)
30	138.3		19.2	0.83 (3H, d, 6.6)	27.0	1.70 (3H, s)
31	31.2	1.81 (1H, m)	17.6	1.36 (3H, d, 7.2)		
32	18.6	0.74 (3H, d, 6.6)				
33	17.5	0.76 (3H, d, 6.6)				
2-NH		8.45 (1H, br s)				
19-OH		5.66 (1H, s)				
15-NH				4.01 (1H, dd, 7.2, 7.2)		
21-NH		3.13 (1H, overlap)				
27-OH				6.56 (1H, d, 4.8)		
27-CONH_2_						8.36 (1H, br s)
						8.66 (1H, br s)

*Aspertoryadin I (****2****):* yellow powder solid; [α]25 D −144 (*c* 0.1, MeOH); UV (MeOH) λ_*max*_ (log ε): 205 (3.6), 226 (3.4), 279 (2.8), and 309 (2.5) nm; ECD (MeOH) λ_*max*_ (Δε): 200 (2.59), 210 (27.82), 231 (−22.02), 316 (−0.95) nm; IR (KBr) υ_*max*_ (cm^–1^): 3,354 (−OH), 2,965 (−CH), 2,926 (−CH), 1,677(−C=O), 1,602 (−NH), 1,475(-C-N), and 1,066 (−C-C-); ^1^H NMR and ^13^C NMR data, see Table 1; HRESIMS *m/z* [M − H]^–^ 473.1833 (calcd for C_26_H_25_N_4_O_5_, 473.1830).

*Aspertoryadin J (****3****):* yellow powder solid; [α]25 D +32 (*c* 0.1, MeOH); UV (MeOH) λ_*max*_ (log ε): 206 (3.8), 228 (3.7), 276 (3.1), and 304 (3.9) nm; ECD (MeOH) λ_*max*_ (Δε): 219 (−5.83), 230 (−47.81), 300 (−4.07), 320 (−2.66), 360 (−0.14); IR (KBr) υ_*max*_ (cm^–1^): 3,412 (−OH), 2,925 (−CH), 1,678 (−C=O), 1,474 (−C−N), and 1,027(−C-C-); ^1^H NMR and ^13^C NMR data, see Table 1; HRESIMS *m/z* [M − H]^–^ 486.1421 (calcd for C_2__5_H_2__0_N_5_O_6_, 486.1419).

### Computational Section

The initial conformational search was carried out in Confab (O’Boyle et al., 2011) using the MMFF94 molecular mechanics force field. Density functional theory calculations were performed using the Gaussian 16 package (Frisch et al., 2019). These conformers were optimized at B3LYP/6-31G (d) in the gas phase, and the conformers with a population over 1% were kept. Then, these conformers were further subjected to geometry optimizations at B3LYP/6-311G (d) in the gas phase, and frequency analysis of all optimized conformations was also performed at the same level of theory to exclude the imaginary frequencies. NMR shielding tensors were calculated with the gauge-independent atomic orbital (GIAO) method at mPW1PW91/6-311G (d,p) level with the IEFPCM solvent model in DMSO. The shielding constants obtained were converted into chemical shifts by referencing to TMS at 0 ppm (δcal = σTMS – σcal), where the σTMS was the shielding constant of TMS calculated at the same level. For each candidate, the parameters a and b of the linear regression δcal = *a*δexp + *b*; the correlation coefficient, *R*^2^; the mean absolute error (MAE) defined as Σ*n* |δcal – δexp| /*n*; the corrected mean absolute error (CMAE), defined as Σ*n* |δcorr – δexp|/*n*, where δcorr = (δcal – *b*)/*a*, were calculated. DP4+ probability analysis was performed using the calculated NMR shielding tensors (Grimblat et al., 2015). The ECD spectra were calculated by the TDDFT methodology at the B3LYP/TZVP utilizing IEFPCM in methanol. ECD spectra were simulated using SpecDis 1.71 (Bruhn et al., 2013) with σ = 0.30 eV.

### α-Glucosidase Inhibition Assay

Yeast α*-glucosidase* (0.2 U/mL) in 0.1 M phosphate buffer (pH 6.8), was used as an enzyme source. The substrate solution of *p*-nitrophenyl-α-D-glucopyranoside (PNPG, 2.5 mM), was prepared in 0.1 M phosphate buffer (pH 6.8) solution. The tested compounds were prepared at different concentrations (250, 500, 1,000, 2,000, and 4,000 μg/mL) in DMSO. The tested compound solution (10 μL) was pre-incubated with 100 μL α-glucosidase solution for 15 min. After pre-incubation, 45 μL of the substrate was added and further incubated for another 15 min at room temperature. The absorbance of each well was measured in Microplate Reader (Thermo Fisher Scientific, United States) reader at 405 nm. All experiments were carried out in triplicate (Dan et al., 2019).

### Homology Modeling of α-Glucosidase

Since there is no structural information of α-glucosidase, we model the homology of it by modeler 9.20 (Webb and Sali, 2016). The amino acid sequence of α-glucosidase AL12 from Baker’s yeast was retrieved from UniProt protein knowledgebase (accession number P53341). The crystal structure of isomaltase (PDBID:3A4A), with 75% sequence identity with the target sequence as the template for homology modeling, was selected after a search in the Protein Data Bank (PDB) at National Center or Biotechnology and Information (NCBI) using BLAST. The constructed model was validated by Procheck, ERRATE, and verify3D programs (Laskowski et al., 1993; Eisenberg et al., 1997).

### Molecular Docking

The 3D structures of the active compounds **10** and **16** were constructed and minimized by MM2 force field using Chemoffice 14.0 software. All hydrogen atoms and gasteiger charges were added to modeled receptor by AutoDock Tools. Docking was performed centered at the active pocket of the α-glucosidase with Autodock vina software (Trott and Olson, 2010). The poses were ranked by their binding affinities and the lowest one was selected as the predicted protein–ligand complexes. The results were presented by Pymol^1^.

### Time-Lapse [Ca^2+^]_*ER*_ Measurements

The biological activity of compounds against the insect ryanodine receptor (RyR) was tested using HEK cells stably expressing RyR from *Spodoptera frugiperda* (sfRyR) or RyR1 from rabbit (rRyR1), and R-CEPIA1er, an engineered endoplasmic reticulum (ER)-targeting fluorescent protein used to measure ER luminal Ca^2+^ concentration ([Ca^2+^]_*ER*_) (Suzuki et al., 2014). Cells were cultured in Dulbecco’s modified Eagle medium containing 10% fetal bovine serum, 15 mg/mL blasticidin, 100 mg/mL hygromycin, and 400 mg/mL G418 first in Petri dishes, and later seeded into 96-well plates at a density of 10^4^ cells/well. After seeding for 24 h, 2 mg/mL doxycycline was added to induce the expression of RyR. After 48 h of induction, the medium was replaced by HEPES-buffered Krebs solution, and [Ca^2+^]_*ER*_ was measured using FlexStation 3 fluorometer (Molecular Devices) by monitoring the fluorescence signal changes. The R-CEPIAer signal, which is excited at 560 nm and emitted at 610 nm, was captured every 10s for 300s. The compounds for screening were added 100s after the recording started. The ratio of the average fluorescence for the last 100s (F) and first 100s (F0), F/F0, was used to report the fluorescence change caused by the compounds. All experiments were carried out in triplicate and repeated twice.

## Results and Discussion

### Structure Elucidation

**FIGURE 2:**
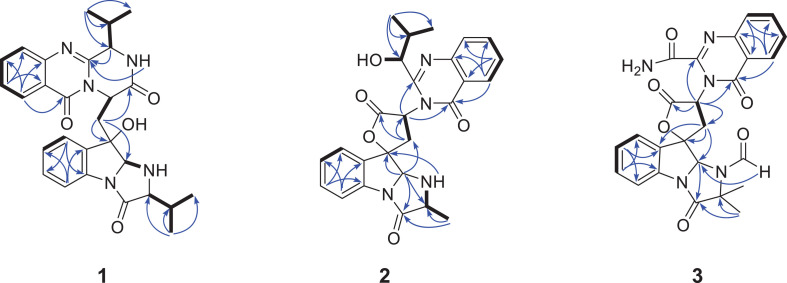
The key HMBC (arrows) and COSY (bold) correlations for compounds **1**–**3**.

**FIGURE 3:**
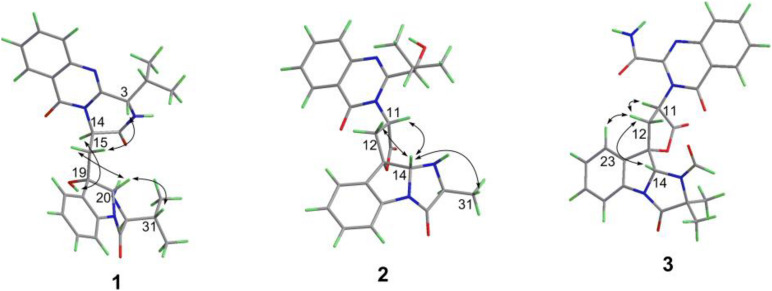
Key ROESY correlations (double arrows) of compounds **1**–**3**.

**FIGURE 4:**
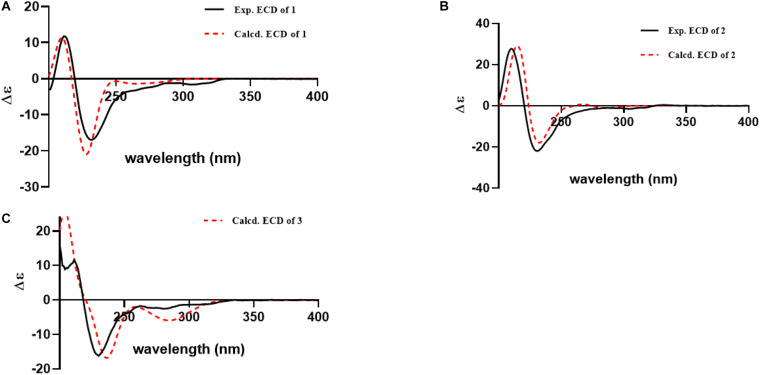
Experimental and calculated ECD curves for compound 1 **(A)**; compound 2 **(B)**; compound 3 **(C)**.

Compound **1** was obtained as yellow powder solid, and its molecular formula was determined to be C_28_H_31_N_5_O_4_ on the basis of HRESIMS, indicating 16 degrees of unsaturation. The ^1^H NMR spectrum (Table 1) showed the obvious resonances for eight aromatic protons attributed to two disubstituted benzene rings and four methyl protons. Its ^13^C, DEPT, and HSQC NMR data (Table 1) revealed a total of 28 carbons, including four methyls, one methylene, 14 methines (six sp^3^ and eight olefinic), and nine quaternary carbons (including three carbonyl groups and five olefinic). A detailed comparison of the NMR data of **1** with those of *epi*-fiscalin C (Buttachon et al., 2012) indicated that they shared the same skeleton with the only difference being the presence of an isopropyl at C-22 in **1** instead of two methyls in *epi*-fiscalin C. The key ^1^H-^1^H COSY cross-peaks of H-22/H-31/H-32 (H-33) and the HMBC correlations from H_3_-32 and H_3_-33 to C-31 and C-22 confirmed the assignment of the isopropyl at C-22. Thus, the planar structure of **1** was established as shown in Figure 2. The relative configuration of the quinazoline and 5/5/6 tri-cyclic rings (Figure 3) were determined by the rotating frame overhauser effect spectroscopy (ROESY) spectrum, in which the correlations of H-20 with H-15a (δ_*H*_ 2.67) and H-31 revealed that H-20, H-31, and CH_2_-15 were on the same face of the 5/5/6 tri-cyclic ring system. The ROESY correlation between H-3 and H-15b indicated that H-3 and C-15 should be placed on the same face of the quinazoline ring. Due to the flexibility of the single bonds C14-C15 and C15-C19, the relative configuration of **1** could not be clearly determined by ROESY spectrum. Therefore, a theoretical NMR calculation with DP4+ analysis was applied to clarify the relative configuration of **1**. The chemical shifts of two isomers (3*S*,14*R*,19*S*,20*R*,22*S*)-**1** and (3*R*,14*S*,19*S*,20*R*,22*S*)-**1**, were predicted using the GIAO method. DP4+ probability analysis showed that (3*S*,14*R*,19*S*,20*R*,22*S*)-**1** was the most likely candidate structure, with a 100% DP4+ (all data). As for the absolute configuration of **1**, the ECD spectrum of (3*S*,14*R*,19*S*,20*R*,22*S*)-**1** was calculated, the result of which matched well with an experimental curve (Figure 4A), establishing the absolute configuration of **1** as presented in Figure 1.

Compound **2** was isolated as yellow powder solid and had the molecular formula C_26_H_26_N_4_O_5_ as established by HRESIMS data with 16 degrees of unsaturation. Its ^13^C (Table 1), DEPT, and HSQC NMR spectra revealed a total of 26 carbons including 13 aromatic carbons (eight protonated), one sp^3^ methylene, three carbonyl carbons, five sp^3^ methine, three methyls, and one oxygenated sp3 quaternary carbon. The above data were similar to those of aspertoryadin A (Kong et al., 2019), indicating that they had a similar skeleton. The obvious structural difference between them is that a hydrogen on N-15 in **2** replaced the methyl sulfonyl group in aspertoryadin A. In addition, there are two methyls at C-16 in aspertoryadin A, but one methyl in **2**. The above deduction was supported by the contiguous COSY cross-peaks (Figure 2) of H-14/NH-15/H-16/H-31 and the key HMBC correlations (Figure 2) from H_3_-31 and H-14 to C-16 and C-17. The relative configuration of **2** was established by ROESY spectrum. The ROESY cross-peaks (Figure 3) of H-11/H-14/H-12a and H-14/H_3_-31 led to the assignment of the relative configurations for the stereocenters C-13, C-14, C-11, and C-16. The stereocenter C-27 was far away from other stereocenters and no ROE correlation was available for the assignment of its relative configuration. Thus, the chemical shifts of two isomers (11*S*,13*S*,14*R*,16*S*,27*S*)-**2** and (11*S*,13*S*,14*R*,16*S*,27*R*)-**2**, were calculated and the former showed 100% DP4+ (all data) probability, enable assignment of 27*S*^∗^ configuration for **2**. The absolute configuration of **2** was confirmed as 11*S*,13*S*,14*R*,16*S*,27*S* by comparison of its experimental ECD spectrum with the calculated ECD curves of **2** (Figure 4B).

Compound **3**, a yellow powder solid, was assigned the molecular formula C_2__5_H_2__1_N_5_O_6_ by its HRESIMS, requiring 18 degrees of unsaturation. The ^13^C NMR and DEPT spectra displayed five carbonyl carbons (including an aldehyde carbon and four amide or ester carbonyls), 13 aromatic carbons (eight protonated), one sp^3^ methylene, two sp^3^ methines, and two sp^3^ quaternary carbons (one oxygenated). These data were also indicative of a quinazoline-containing indole alkaloid skeleton as that of **2**. A direct comparison of the NMR data of **3** with those of **2** revealed that the differences between them are the presence of an additional aldehyde group and one additional methyl group at the N-15 and C-16 in **3**, respectively, as well as the attachment of an amide moiety in **3** instead of an isobutyl unit at C-3 as in **2**. The additional groups were supported by the HMBC correlations (Figure 2) from H-28 (δ_*H*_ 8.64) to C-14 (δ_*C*_ 81.5) and from H_3_-29 and H_3_-30 to C-16, and C-17. The ROESY cross-peaks of H-12a/H-14/H-11 and H-23/H-12b indicated that **3** possessed the same relative configurations at C-11, C-13, and C-14 as aspertoryadin A (Figure 3). The absolute configuration was determined to be 11*S*, 13*S*, and 14*R* by ECD calculation (Figure 4C).

### α-Glucosidase Inhibition Assay

All of the compounds isolated were evaluated for α-glucosidase inhibitory activity (Apostolidis et al., 2007). Compounds **10** and **16** exhibited potent α-glucosidase inhibitory activities with the IC_50_ values of 7.18 and 5.29 μM, respectively (Acarbose as a positive control, IC_50_: 213.0 μM).

### Molecular Docking

**FIGURE 5:**
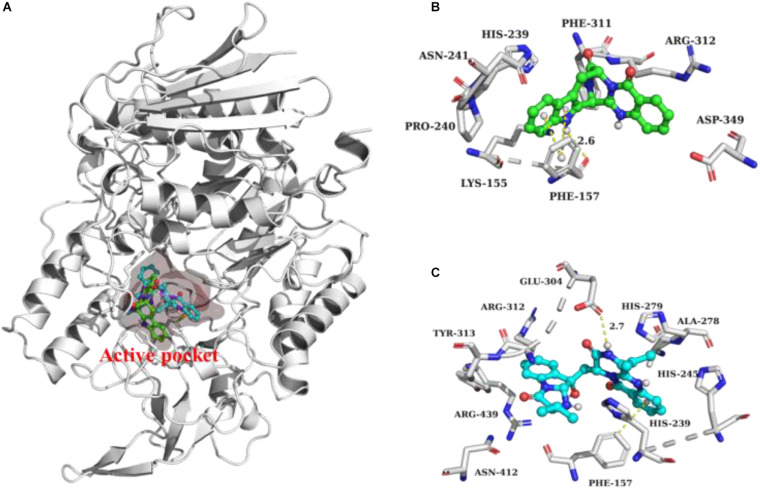
Docking analysis of **10** (green ball and stick) and **16** (cyan ball and stick) with α-glucosidase **(A)**. 3D cartoon diagram of the interactions of **10 (B)** and **16 (C)** with α-glucosidase.

The intermolecular interaction and potential binding sites between compounds **10**, **16,** and α-glucosidase were investigated via molecular docking simulations. The docking simulation results (Figure 5) demonstrated that compound **10** could interact with α-glucosidase by forming one hydrogen bond and π–π interactions with residues PHE 157 (Figure 5B). While compound **16** could generate one hydrogen bond with residues GLU 340 and also form π–π interactions with residues PHE 157 in α-glucosidase (Figure 5C).

### Biological Activity Against RyR

**FIGURE 6:**
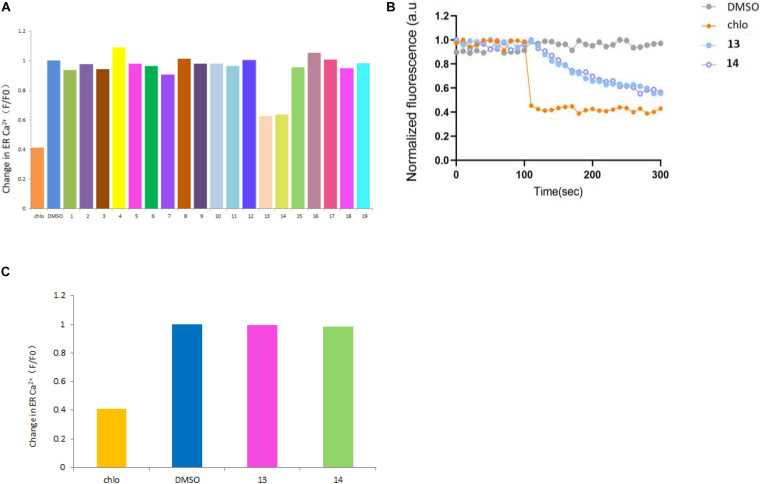
Summary of against activities of compounds **1**–**19** on sfRyR **(A)** and compounds **13** and **14** on rRyR1 **(C)** at 100 μM; Time-lapse R-CEPIA1er fluorescence measurement curves of **13** and **14 (B)** with chlorantraniliprole (chlo) and DMSO as positive and negative controls, respectively.

Ryanodine receptor, an intracellular calcium channel located on ER membrane, is a well-known insecticide target. The top-selling diamide insecticides, such as flubendiamide, chlorantraniliprole, and cyanotraniliprole, all target insect RyRs. The insecticidal activities of all compounds were tested against RyRs from an agricultural pest, *S. frugiperda*, using time-lapse [Ca^2+^]_*ER*_ measurements. At 100 μM concentration, compounds **13** and **14**, showed a clear activation effect against sfRyR, which reduced the [Ca^2+^]_*ER*_ by 37.1 and 36.2%, respectively (Figure 6A). While their effect in intracellular Ca2 release is similar to that of the positive control chlo, the release rate is much slower (Figure 6B) suggesting different binding sites and different mechanisms of action on the sfRyR. Compounds **1**, **2,** and **3** also showed some weak activation effects on sfRyR (Figure 6A). The species selectivity of **13** and **14** was further characterized by comparing their activity against rabbit RyR1 (rRyR1). Interestingly, both compounds showed no clear activation activity on rRyR1, suggesting that they can selectively act on insect RyRs and have good potential to be developed into insecticidal molecules (Figure 6C).

## Conclusion

In summary, 19 quinazoline-containing indole alkaloids (**1**–**19**), including 3 new ones, were isolated from the marine-derived fungus *Aspergillus* sp. HNMF114 by supplemented L-tryptophan to its fermentation broth. Among them, compounds **10** and **16** showed α-glucosidase inhibitory activity, that significant activity with potential for further development. Compounds **1**, **2**, and **3** showed weak activities against sfRyR, and **13** and **14** showed moderate activities against sfRyR. Compounds **13** and **14** also have no clear activation activity on rRyR1, which means **13** and **14** could selectively act on insect RyRs and have good potential for the development of insecticidal drugs.

## Data Availability Statement

The original contributions presented in the study are included in the article/Supplementary Material, further inquiries can be directed to the corresponding authors.

## Author Contributions

Y-XZ and D-QL contributed to the conception and design of the study. LYan and F-DK determined the plane structure and absolute configuration. S-SL wrote the first draft of the manuscript and performed all of the experimental work. Q-YM and Q-YX contributed to the isolation of compounds. J-HZ, LYao, and L-MZ contributed to the bioactivity assay. Z-GYC and H-FD improved the manuscript. All authors contributed to manuscript revision as well as read and approved the submitted version.

## Conflict of Interest

The authors declare that the research was conducted in the absence of any commercial or financial relationships that could be construed as a potential conflict of interest.

## Footnotes

1 https://pymol.org
