# Supplementary material for: Three New Quinazoline-Containing Indole Alkaloids From the Marine-Derived Fungus Aspergillus sp. HNMF114
Source: Front Microbiol. 2021 Jun 2;12:680879. doi: 10.3389/fmicb.2021.680879 (PMC8206283; doi:10.3389/fmicb.2021.680879)
Supplement: Supplementary file 1 [file Data_Sheet_1.PDF]

# Supplementary Material

## Three new quinazoline-containing indole alkaloids from the marine-derived fungus *Aspergillus* sp. HNMF114

**Sha-Sha Liu[1,‡], Li Yang[2,‡], Fan-Dong Kong[3, ‡], Jia-Hui Zhao[4], Li Yao[4], Zhi-guang Yuchi[4], Qing-Yun Ma[2], Qing-Yi Xie[2], Li-Man Zhou[3], Meng-Fei Guo[3], Hao-Fu Dai[5,*], You-Xing Zhao[2,*] and Du-Qiang Luo[1,*]**

[1] *College of Life Science, Key Laboratory of Medicinal Chemistry and Molecular Diagnosis of Ministry of Education, Hebei University, Baoding 071002, China;*

[2] *Haikou Key Laboratory for Research and Utilization of Tropical Natural Products, Institute of Tropical Bioscience and Biotechnology, CATAS, Haikou 571101, China;*

[3] *Key Laboratory of Chemistry and Engineering of Forest Products, State Ethnic Affairs Commission, Guangxi Key Laboratory of Chemistry and Engineering of Forest Products, Guangxi Collaborative Innovation Center for Chemistry and Engineering of Forest Products, School of Chemistry and Chemical Engineering, Guangxi University for Nationalities, Nanning 530006, China.*

[4] *Tianjin Key Laboratory for Modern Drug Delivery & High-Efficiency, Collaborative Innovation Center of Chemical Science and Engineering, School of Pharmaceutical Science and Technology, Tianjin University, Tianjin 300000, China.*

[5] *Hainan Institute for Tropical Agricultural Resources, CATAS, Haikou 571101, China.*

## The list of supporting information

1. NMR spectra of 1–3 .......... 1
Figure S1. IR spectrum of Compound 1 (KBr) .......... 1
Figure S2. 1H NMR spectrum of Compound 1 in DMSO-d6 (600 MHz) .......... 1
Figure S3. 13C NMR spectrum of Compound 1 in DMSO-d6 (150 MHz) .......... 2
Figure S4. HRESI (-) MS spectrum of Compound 1 .......... 2
Figure S5. HSQC spectrum of Compound 1 in DMSO-d6 (600 MHz) .......... 3
Figure S6. HMBC spectrum of Compound 1 in DMSO-d6 (600 MHz) .......... 3

Figure S7. COSY spectrum of Compound 1 in DMSO-d6 (600 MHz) ....................................4
Figure S8. ROESY spectrum of Compound 1 in DMSO-d6 (600 MHz)..................................4
Figure S9. IR spectrum of Compound 2 (KBr)..........................................................................5
Figure S10. 1H NMR spectrum of Compound 2 in DMSO- d6 (600 MH z)............................5
Figure S11. 13C NMR spectrum of Compound 2 in DMSO-d6 (150 MHz) ............................6
Figure S12. HRESI (-) MS spectr um of Compound 2 ..............................................................6
Figure S13. HSQC spectrum of Compound 2 in DMSO-d6 (600 MHz) ..................................7
Figure S14. HMBC spectrum of Compound 2 in DMSO-d6 (600 MHz).................................7
Figure S15. COSY spectrum of Compound 2 in DMSO-d6 (600 MHz) ..................................8
Figure S16. ROESY spectrum of Compound 2 in DMSO-d6 (600 MHz)................................8
Figure S17. IR spectrum of Compound 3 (KBr)........................................................................9
Figure S18. 1H NMR spectrum of Compound 3 in DMSO-d6 (600 MHz)..............................9
Figure S19. 13C NMR spectrum of Compound 3 in DMSO-d6 (150 MHz) ..........................10
Figure S20. HRESI (-) MS spectrum of Compound 3 ...........................................................10
Figure S21. HSQC spectrum of Compound 3 in DMSO-d6 (600 MHz) ................................11
Figure S22. HMBC spectrum of Compound 3 in DMSO-d6 (600 MHz)...............................11
Figure S23. COSY spectrum of Compound 3 in DMSO-d6 (600 MHz) ................................12
Figure S24. ROESY spectrum of Compound 3 in DMSO-d6 (600 MHz)..............................12
2. Computational Details..........................................................................................................13
Table S1. The results of the DP4+ analysis.............................................................................13
Figure S25. Linear regression analysis between experimental and calculated 13C NMR chemical shifts of 3*S*,14*R*,19*S*,20*R*,22*S*)-**1** (A) and (3*R*,14*S*,19*S*,20*R*,22*S*) -**1** (B)..........13
Table S2. Experimental and calculated 13C NMR chemical shifts of 14R-1 and 14S-1 ........14
Table S3. Experimental and calculated 1H NMR chemical shifts of 14R-1 and 14S-1..........15
Table S4. Conformational analysis of the optimized isomer 14R-1 and 14S-1 at B3LYP/6-311G(d) level in gas phase. ...........................................................................16
Figure S26. Optimized geometries of isomers 14R-1 and 14S-1 at B3LYP/6-311G(d) level in gas phase ..............................................................................................................16
Table S5. The coordinates for the optimized conformers of 14R-1 in the gas phase at the B3LYP/6-311G(d) level .......................................................................................17
Table S6. The coordinates for the optimized conformers of 14S-1 in the gas phase at the B3LYP/6-311G(d) level .......................................................................................18
Table S7. The DP4+ analysis results of compound 2..............................................................20
Figure 27. Linear regression analysis between experimental and calculated $^{13}$C NMR chemical shifts of (11*S*,13*S*,14*R*,16*S*,27*R*)-**2** (**A**) and (11*S*,13*S*,14*R*,16*S*,27*S* )-**2** (**B**).....21
Table S8. Experimental and calculated 13C NMR chemical shifts of 27R-2 and 27S-2. .......21
Table S9. Experimental and calculated 1H NMR chemical shifts of 27R-2 and 27S-2..........22
Table S10. Conformational analysis of the optimized isomer 27R-2 and 27S-2 at B3LYP/6-311G(d) level in gas phase. ...........................................................................22
Figure S28. Optimized geometries of isomers 27R-2 and 27S-2 at B3LYP/6-311G(d) level in gas phase. ............................................................................................................23
Table S11. The coordinates for the optimized conformers of 27S-2 in the gas phase at the B3LYP/6-311G(d) level. .....................................................................................23
Figure S29. Optimized geometries of isomers 3 at B3LYP/6-311G(d) level in gas phase .....25

Table S12. The coordinates for the optimized conformers of 3 in the gas phase at the B3LYP/6-311G(d) level. ....................................................................................25

## 1. NMR spectra of 1–3

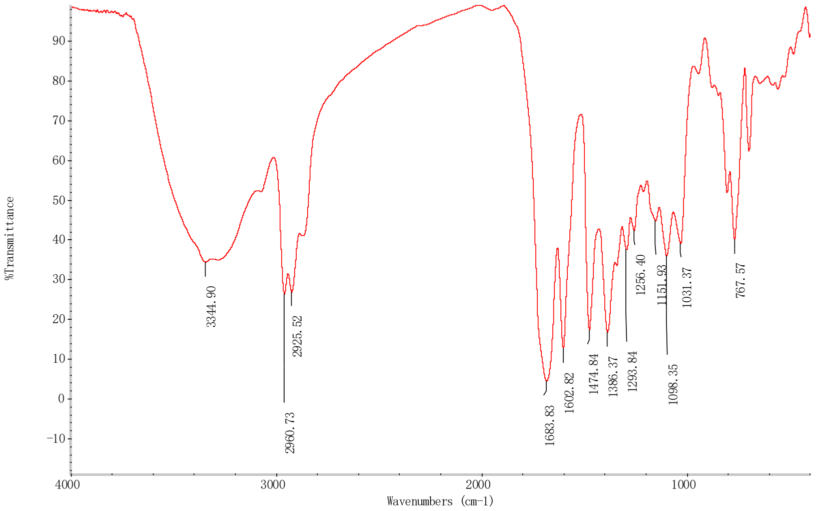


**Figure S1.** IR spectrum of Compound 1 (KBr)

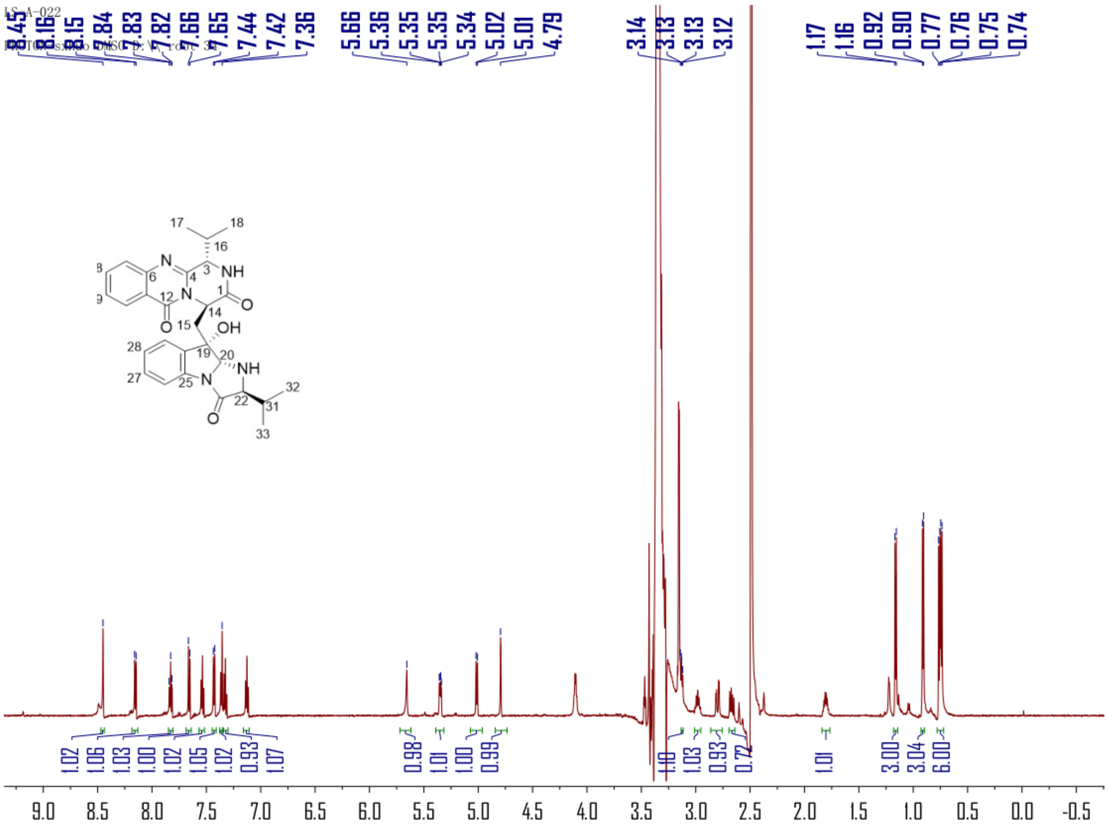


**Figure S2.** $^{1}$H NMR spectrum of Compound **1** in DMSO-$d_6$ (600 MHz)

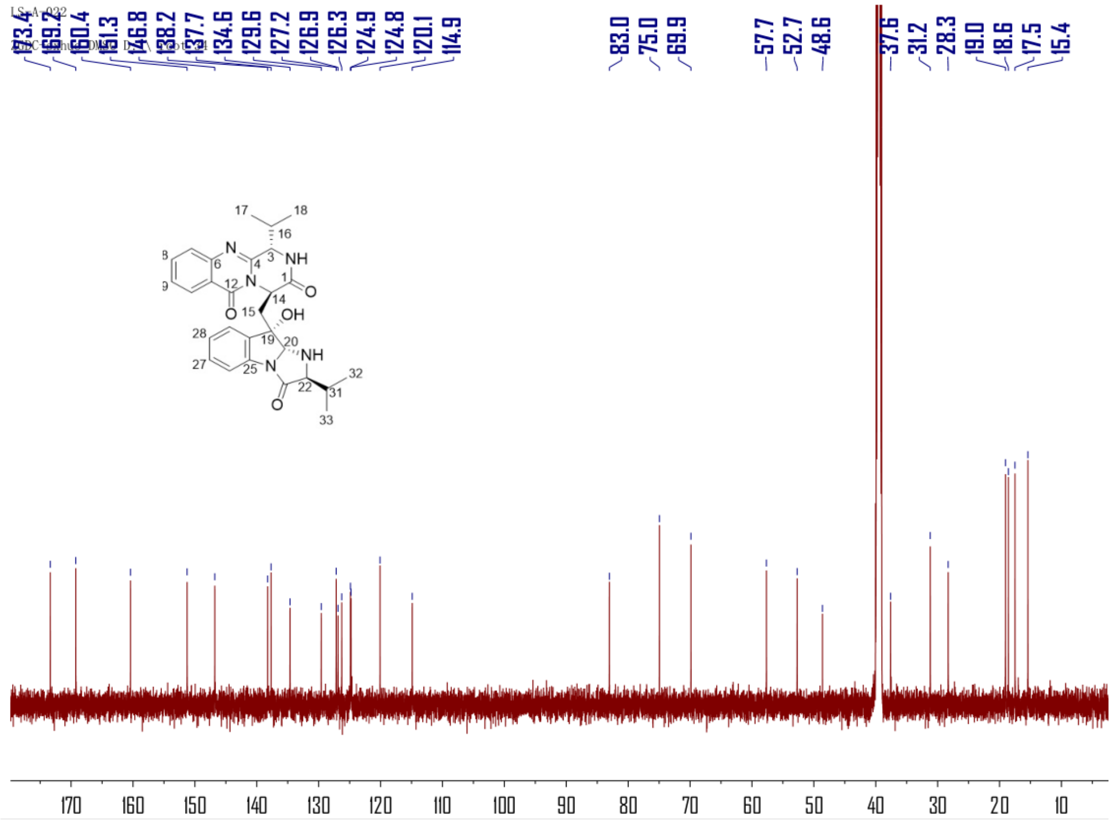


**Figure S3.** $^{13}$C NMR spectrum of Compound **1** in DMSO-$d_6$ (150 MHz)

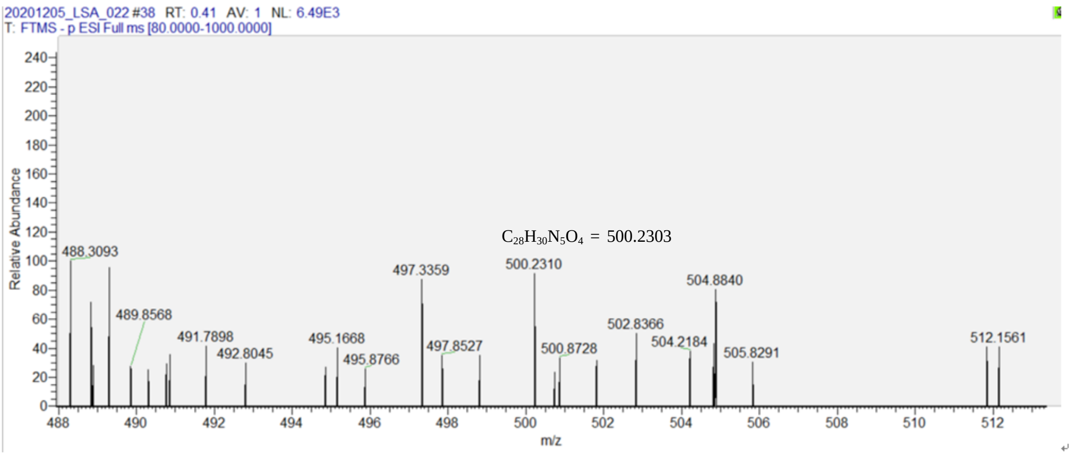


**Figure S4.** HRESI (-) MS spectrum of Compound **1**

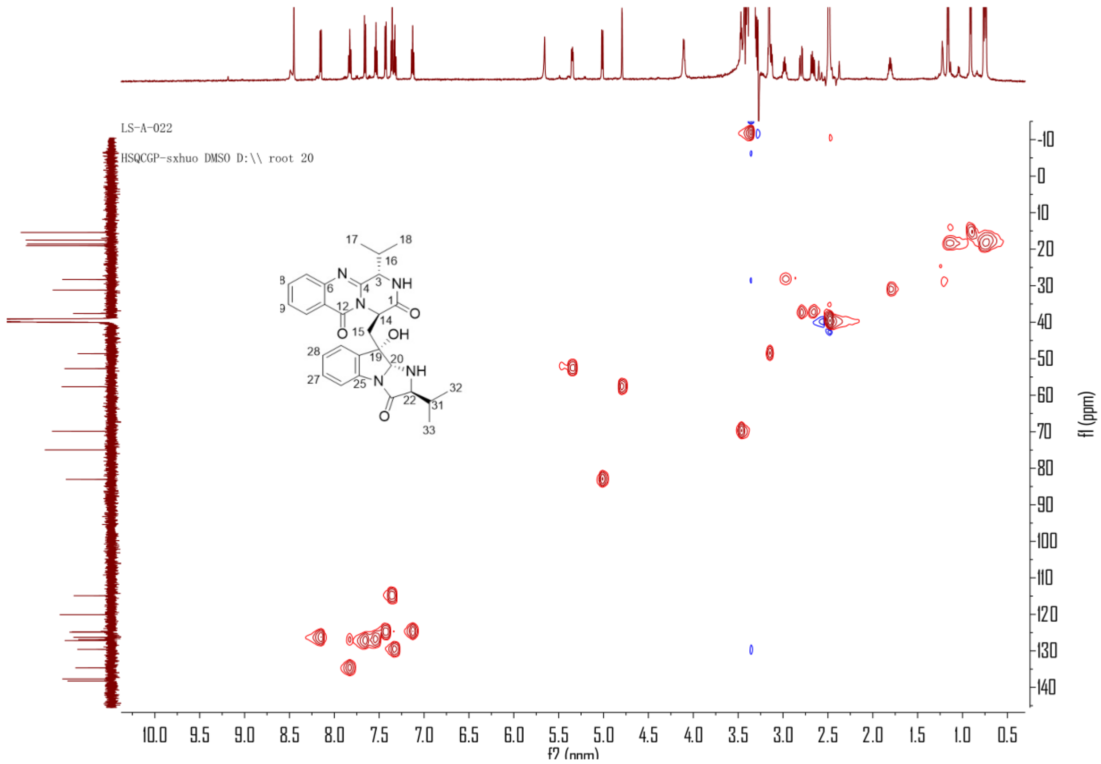


**Figure S5.** HSQC spectrum of Compound **1** in DMSO-$d_6$ (600 MHz)

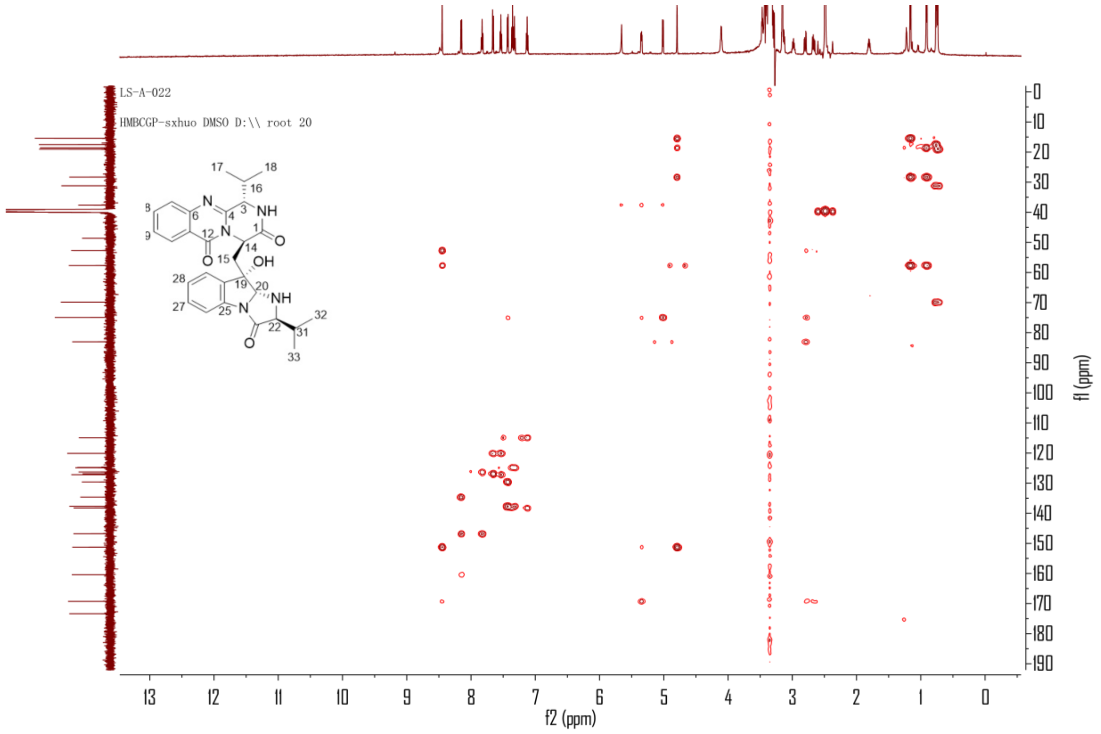


**Figure S6.** HMBC spectrum of Compound **1** in DMSO-$d_6$ (600 MHz)

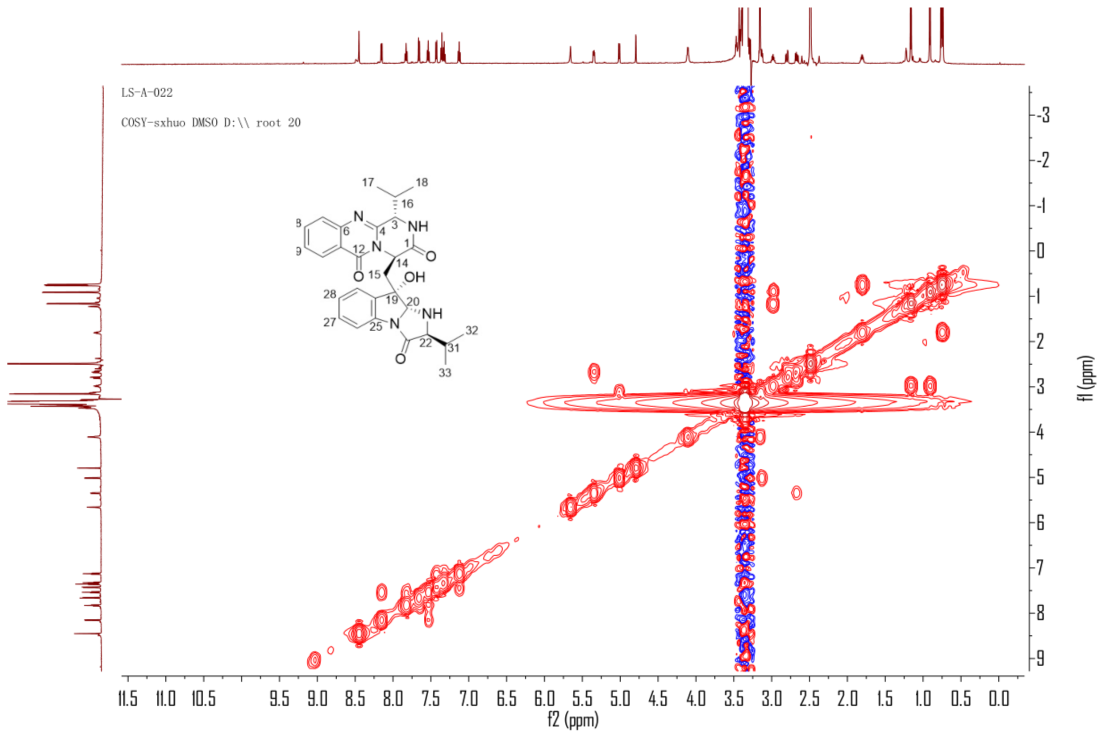


**Figure S7.** COSY spectrum of Compound **1** in DMSO-$d_6$ (600 MHz)

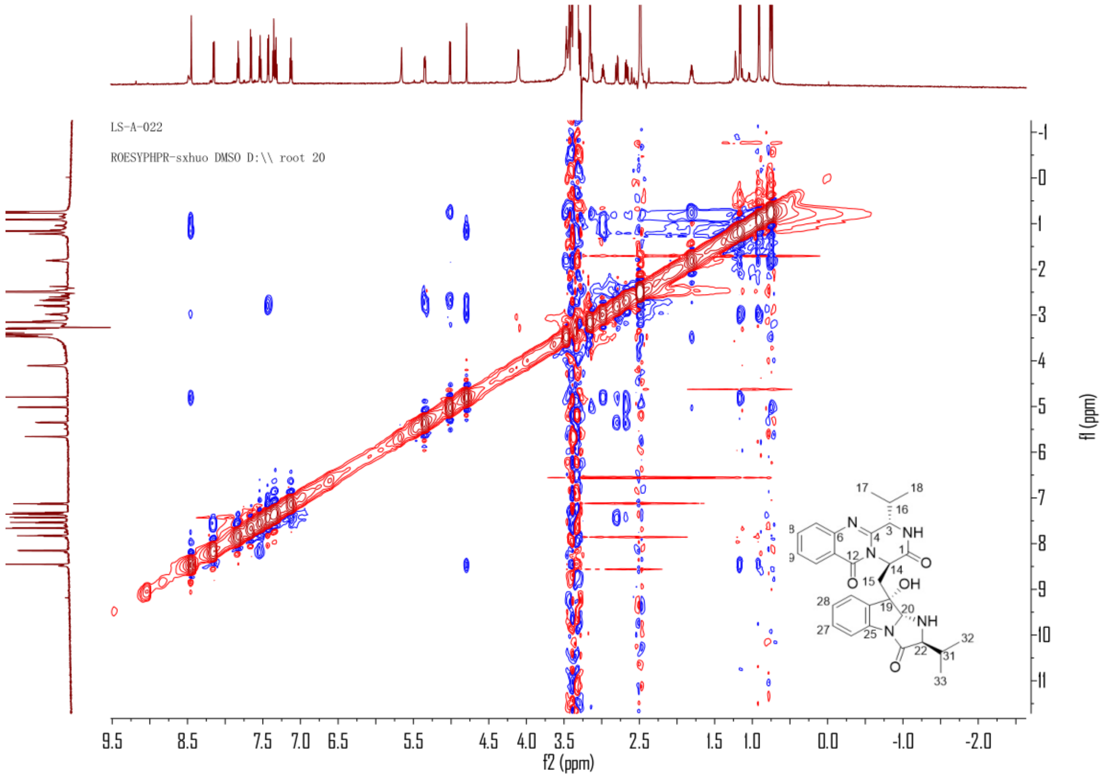


**Figure S8.** ROESY spectrum of Compound **1** in DMSO-$d_6$ (600 MHz)

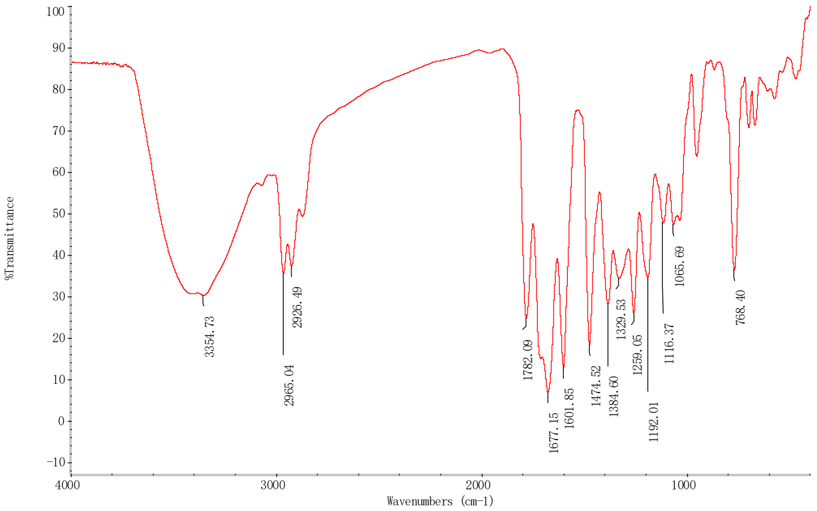


**Figure S9.** IR spectrum of Compound **2** (KBr)

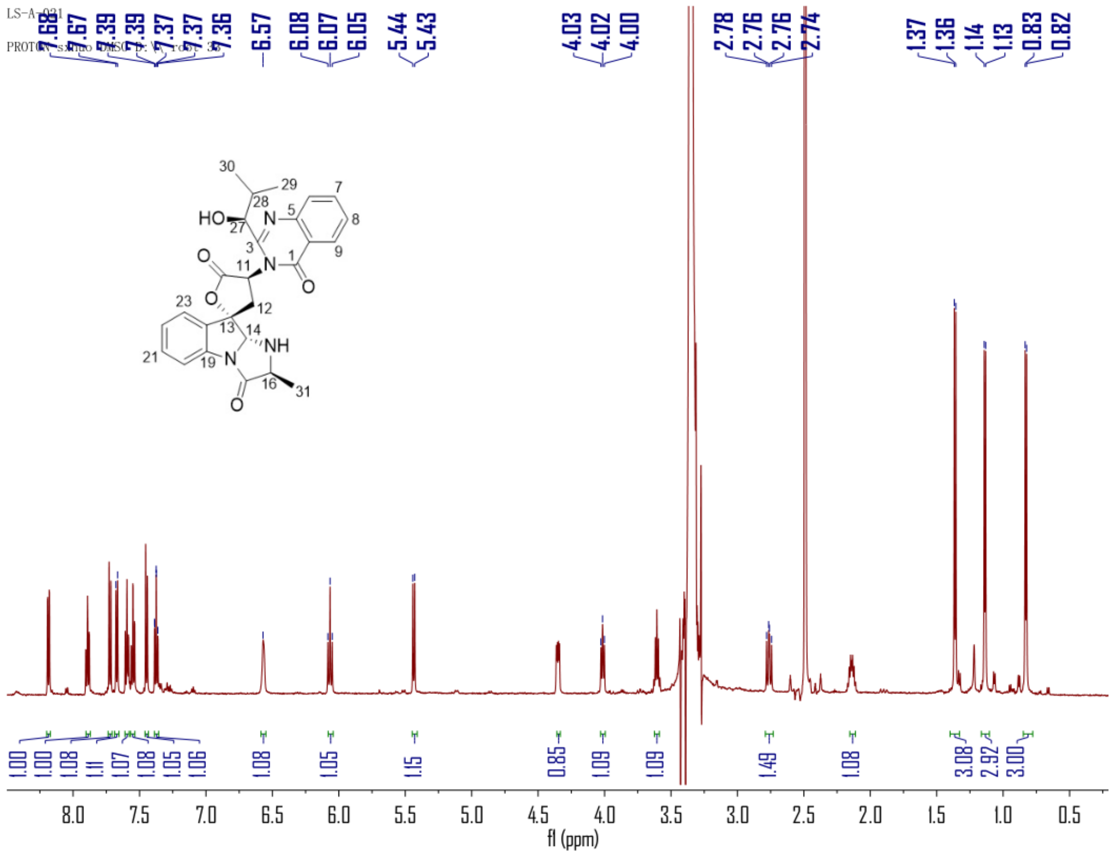


**Figure S10.** $^{1}$H NMR spectrum of Compound **2** in DMSO-$d_6$ (600 MHz)

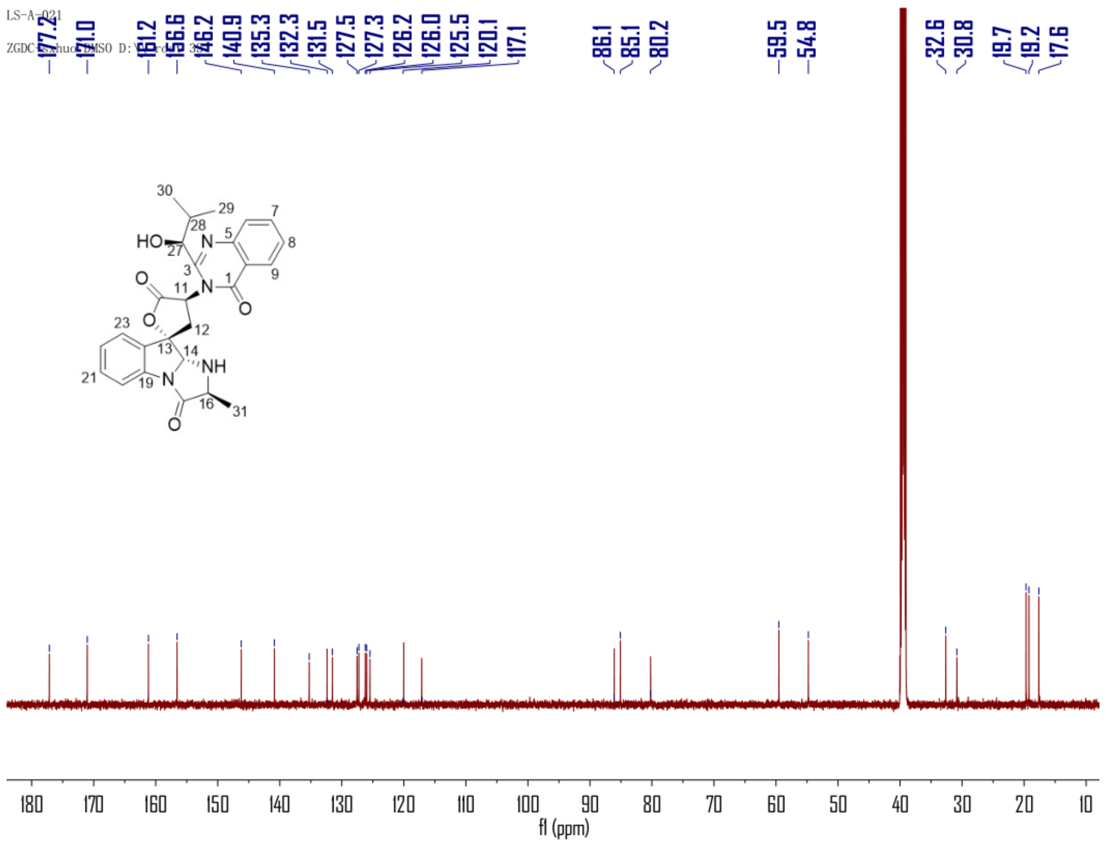

**Figure S11.** $^{13}$C NMR spectrum of Compound **2** in DMSO-$d_6$ (150 MHz)

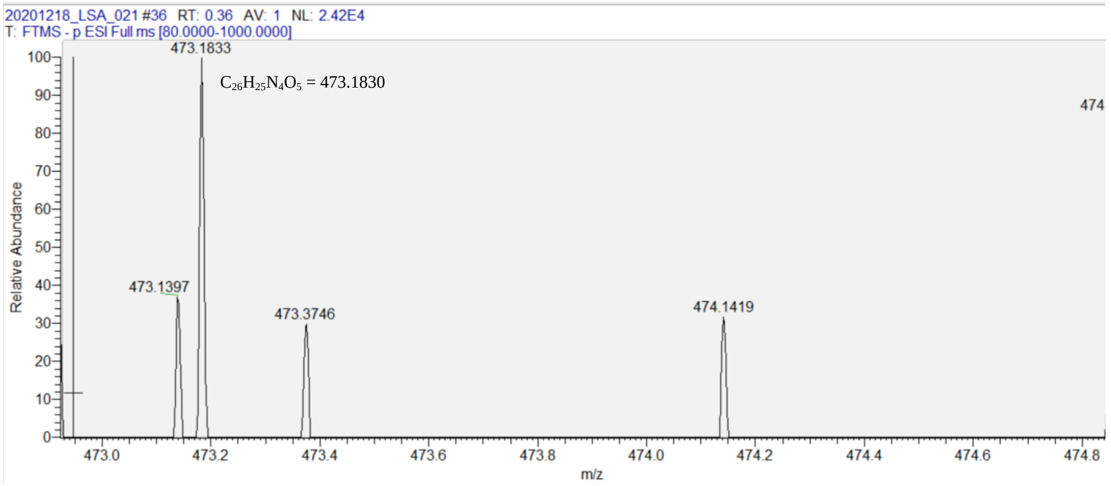

**Figure S12.** HRESI (-) MS spectrum of Compound **2**

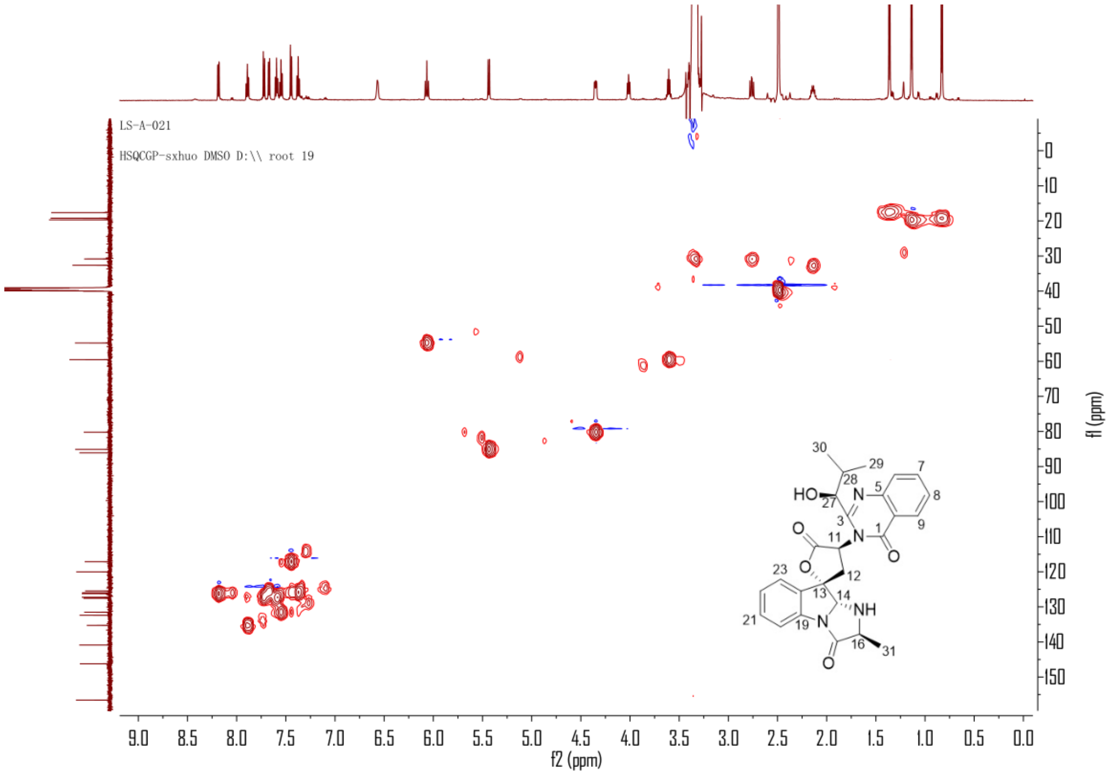


**Figure S13.** HSQC spectrum of Compound **2** in DMSO-$d_6$ (600 MHz)

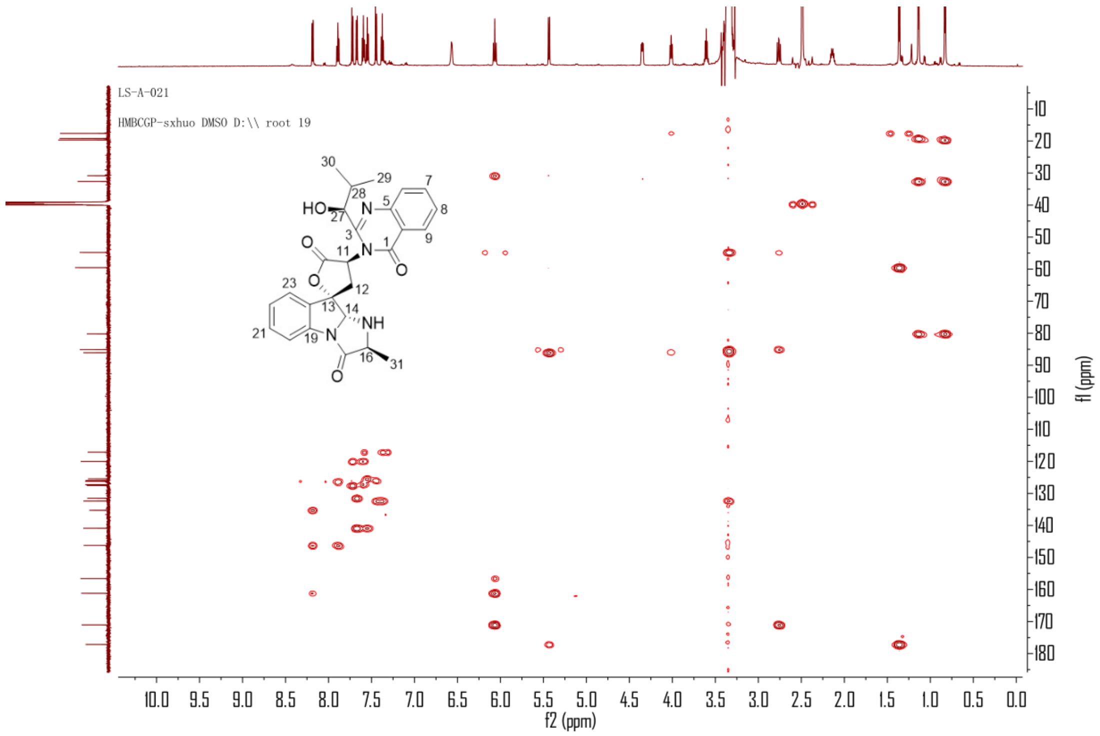


**Figure S14.** HMBC spectrum of Compound **2** in DMSO-$d_6$ (600 MHz)

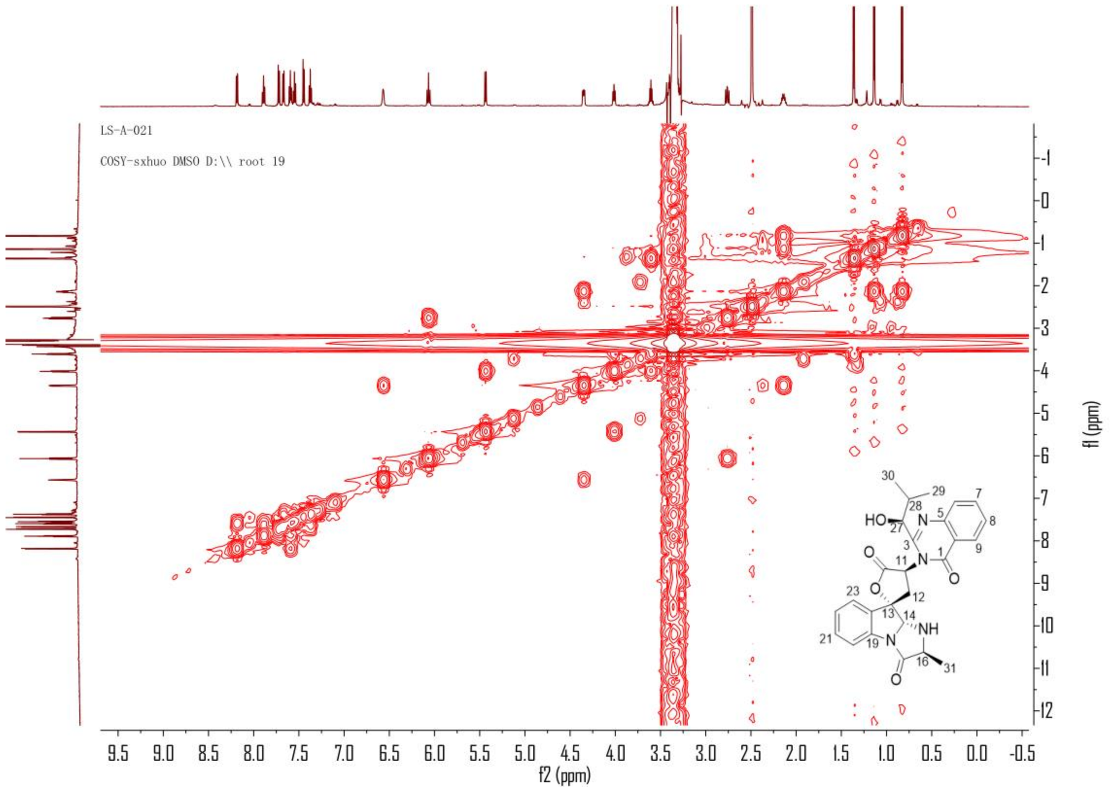


**Figure S15.** COSY spectrum of Compound **2** in DMSO-$d_6$ (600 MHz)

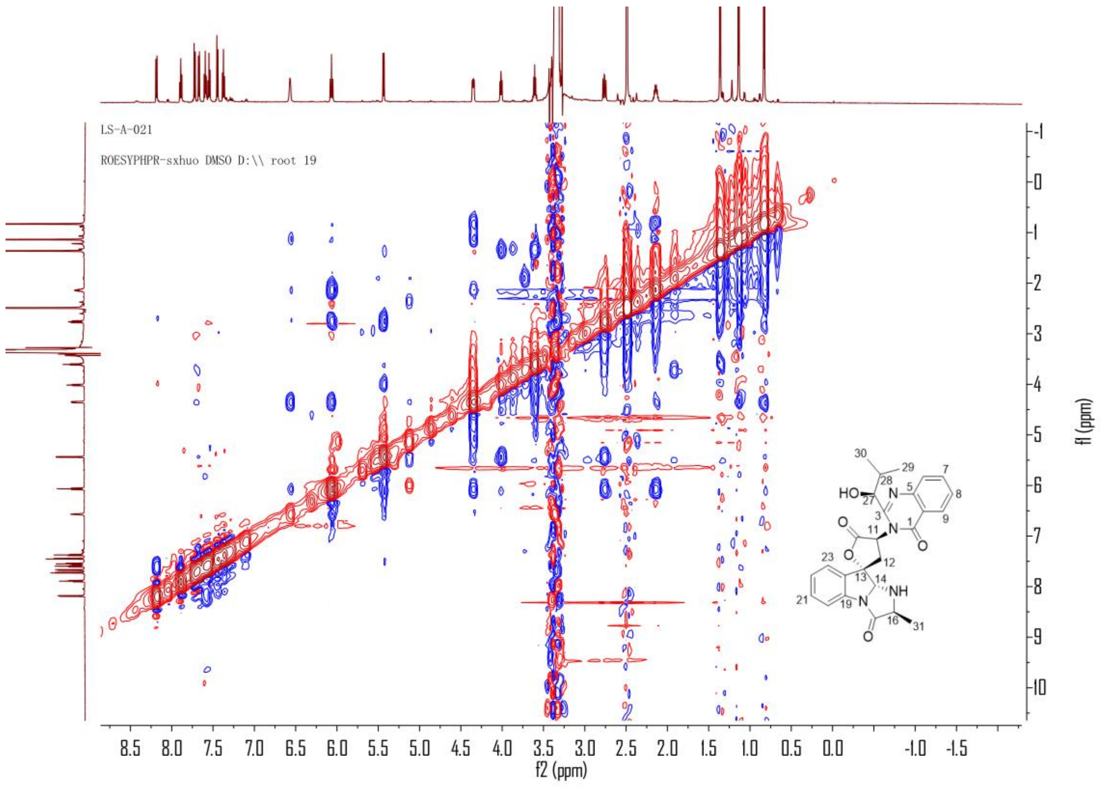


**Figure S16.** ROESY spectrum of Compound **2** in DMSO-$d_6$ (600 MHz)

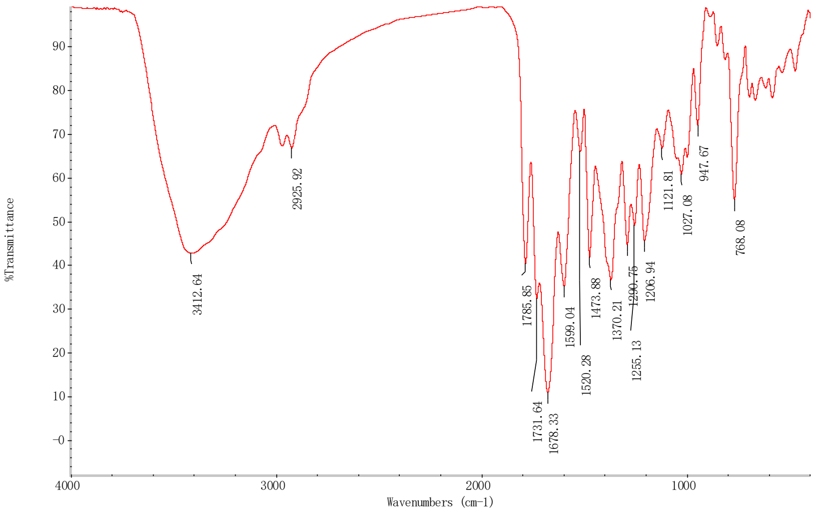

**Figure S17.** IR spectrum of Compound **3** (KBr)

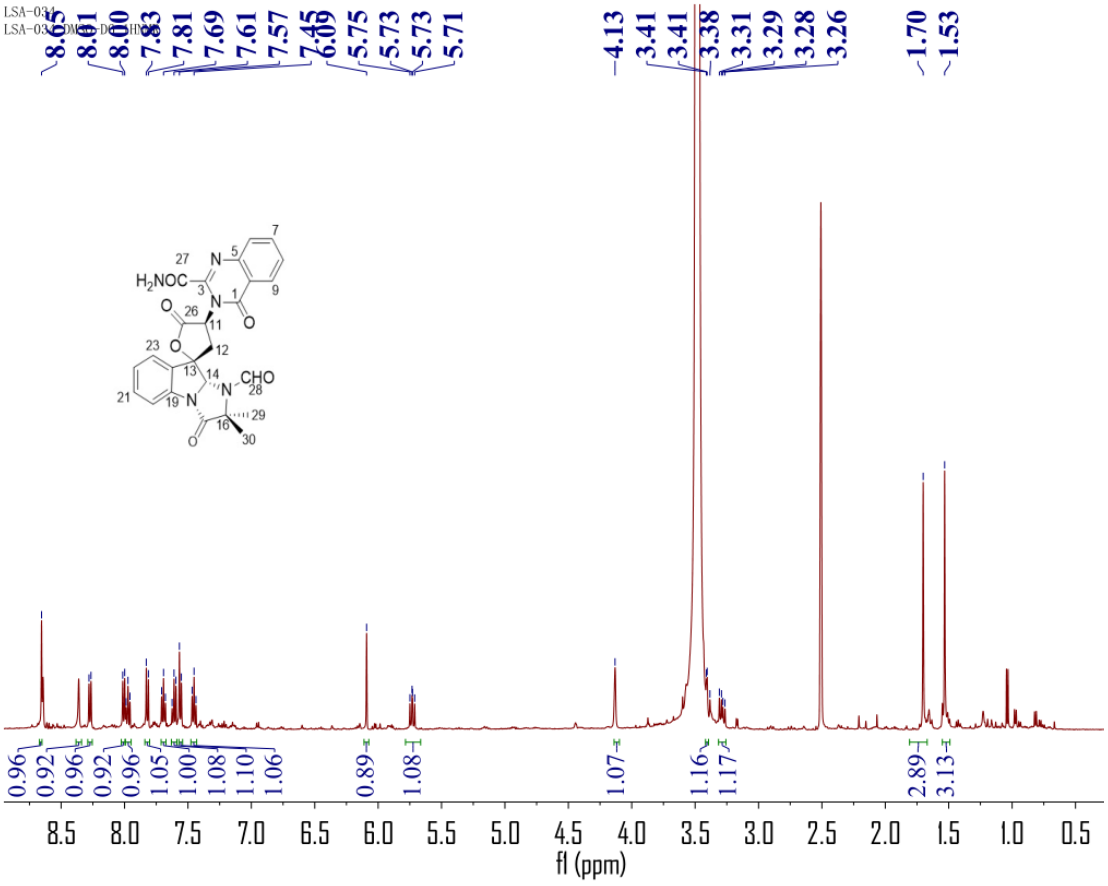

**Figure S18.** $^{1}$H NMR spectrum of Compound **3** in DMSO-$d_6$ (600 MHz)

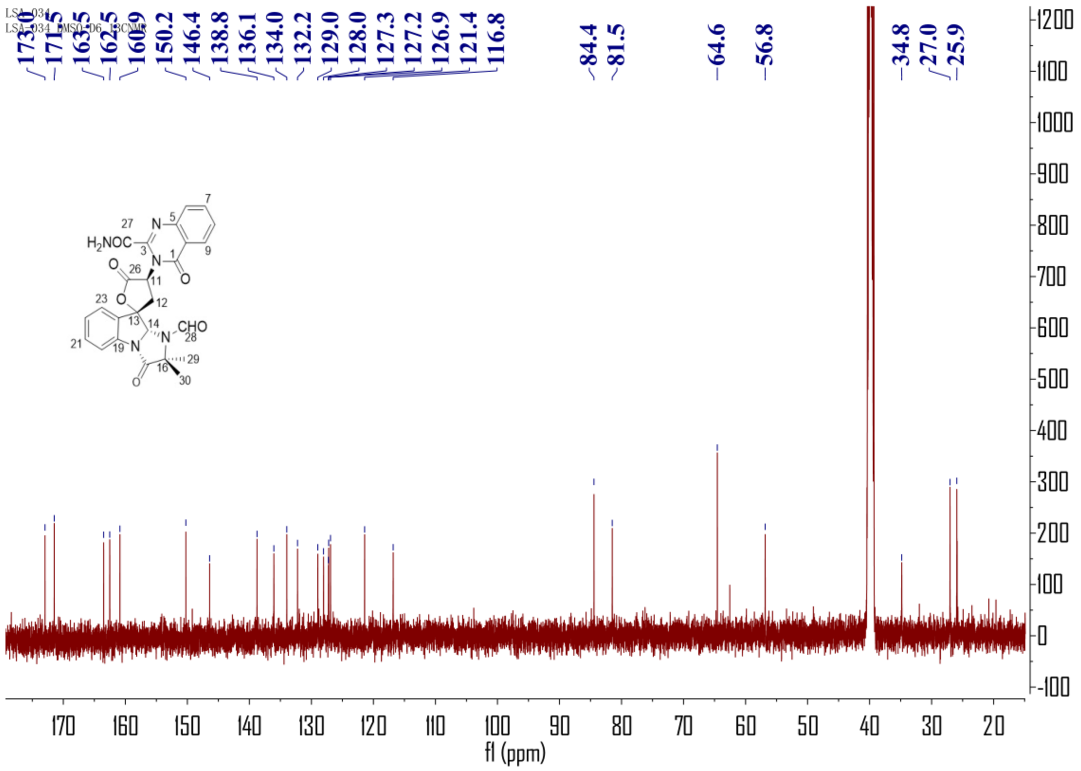


**Figure S19.** $^{13}$C NMR spectrum of Compound **3** in DMSO-$d_6$ (150 MHz)

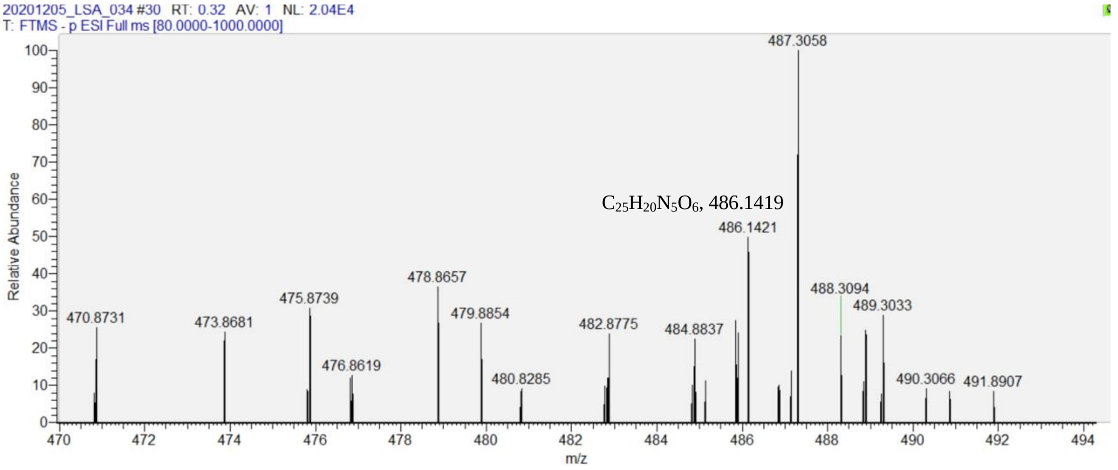


**Figure S20.** HRESI (-) MS spectrum of Compound **3**

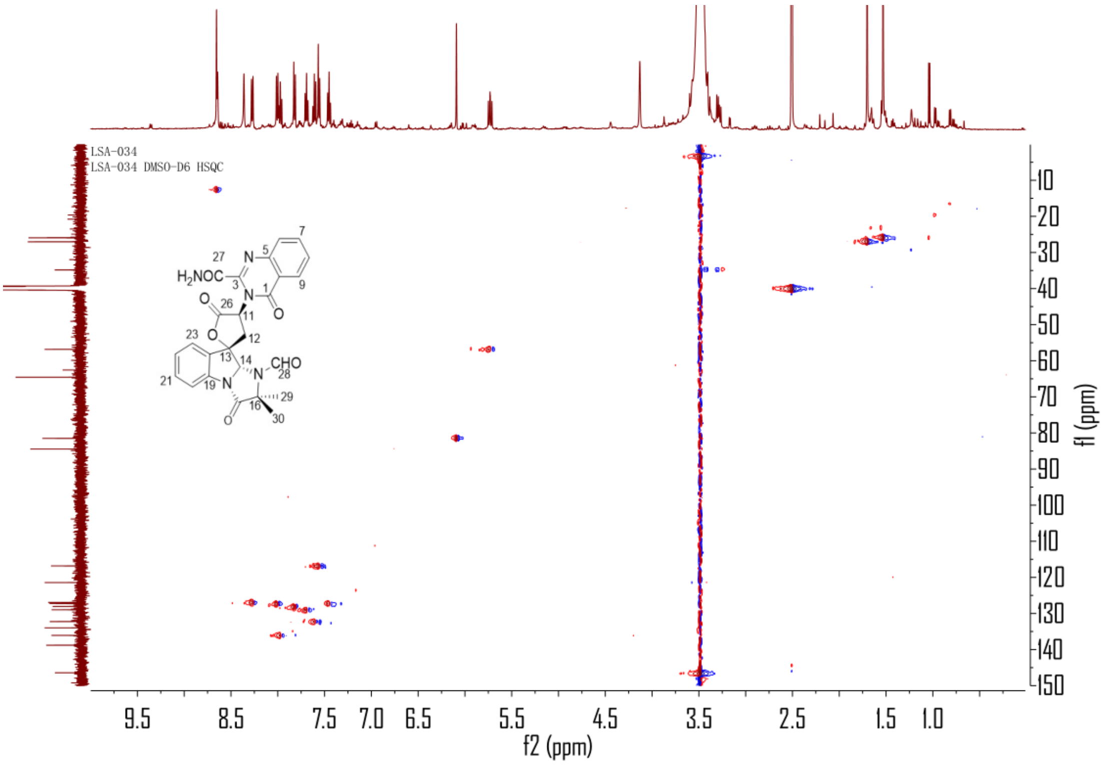


**Figure S21.** HSQC spectrum of Compound **3** in DMSO-$d_6$ (600 MHz)

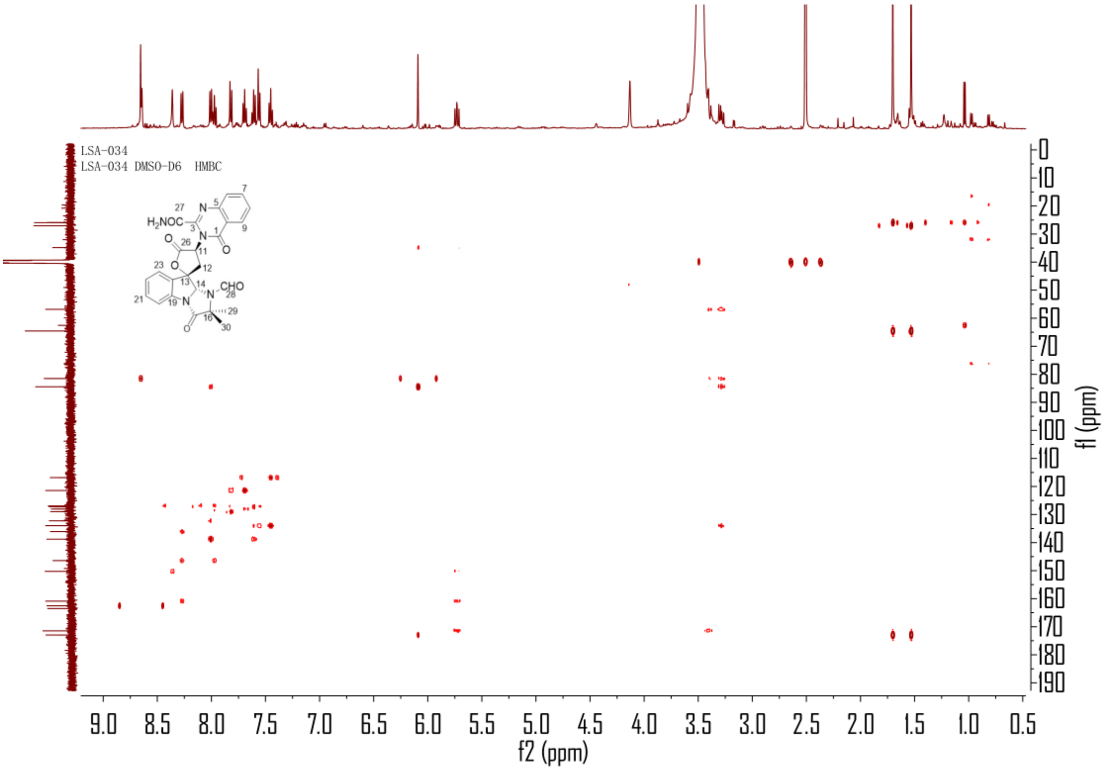


**Figure S22.** HMBC spectrum of Compound **3** in DMSO-$d_6$ (600 MHz)

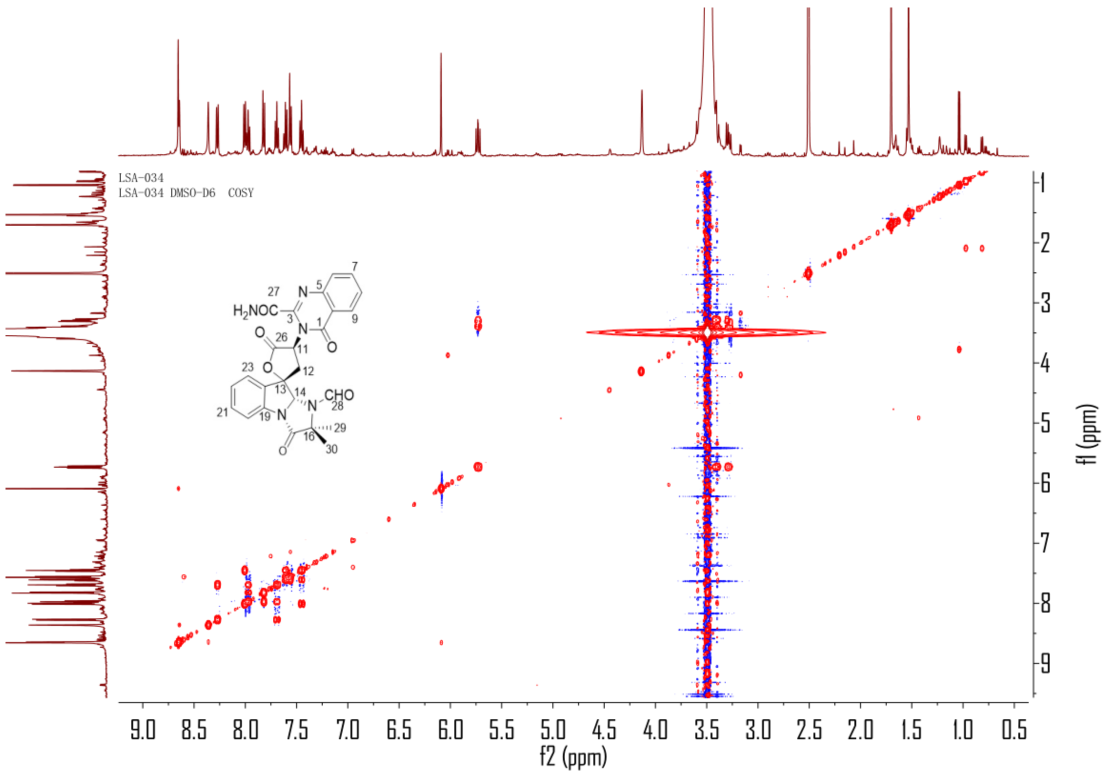


**Figure S23.** COSY spectrum of Compound **3** in DMSO-$d_6$ (600 MHz)

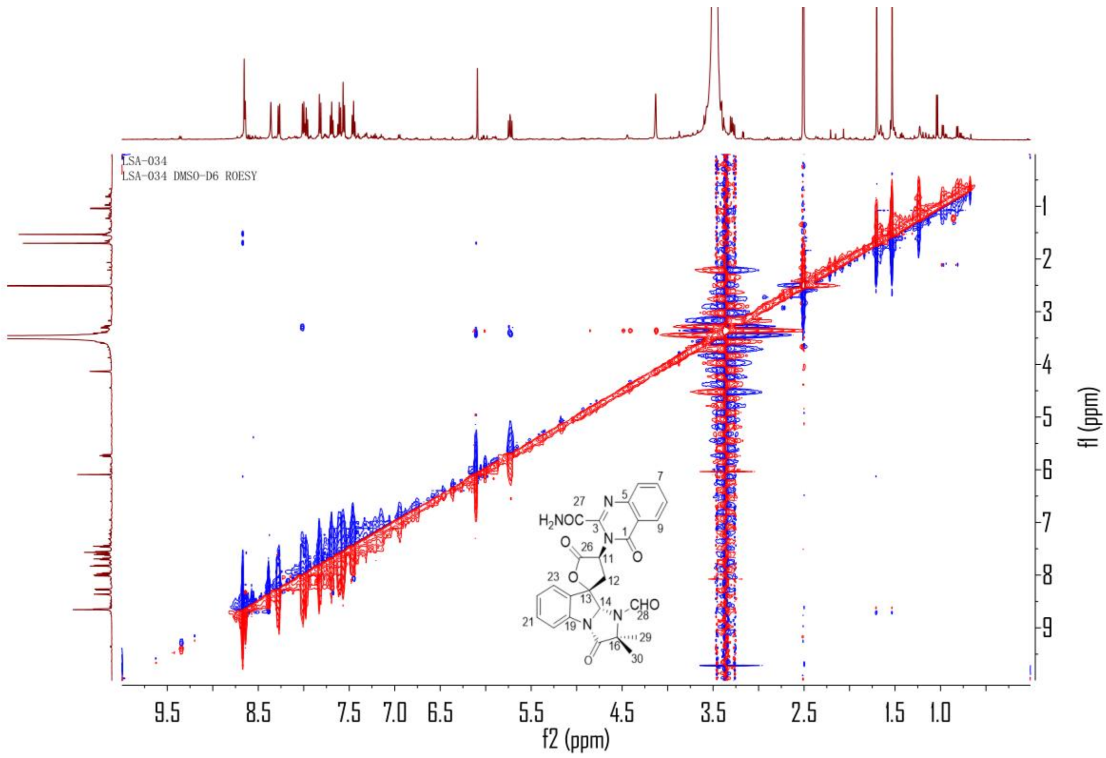


**Figure S24.** ROESY spectrum of Compound **3** in DMSO-$d_6$ (600 MHz)

## 2. Computational Details

**Table S1.** The DP4+ analysis results of compound **1**

| Functional | Solvent? | Basis Set | Type of Data |
|---|---|---|---|
| mPW1PW91 | PCM | 6-311G(d,p) | Shielding Tensors |

| | | DP4+ | 100.00% | 0.00% | - | - | - |
|---|---|---|---|---|---|---|---|
| Nuclei | sp2? | xperimenta | Isomer 1 | Isomer 2 | Isomer 3 | Isomer 4 | Isomer 5 |
| C | x | 160.3 | 14.4 | 15.7 | | | |
| C | | 57.7 | 126.8 | 126.5 | | | |
| C | x | 151.3 | 31.2 | 31.8 | | | |
| C | x | 146.8 | 36.3 | 36.0 | | | |
| C | x | 127.2 | 55.4 | 55.9 | | | |
| C | x | 134.6 | 47.1 | 47.7 | | | |
| C | x | 126.9 | 55.9 | 56.9 | | | |
| C | x | 126.3 | 55.3 | 54.3 | | | |
| C | x | 120.1 | 64.5 | 65.9 | | | |
| C | x | 160.4 | 23.0 | 22.9 | | | |
| C | | 52.7 | 133.8 | 133.6 | | | |
| C | | 37.6 | 148.29 | 150.69 | | | |
| C | | 28.3 | 156.02 | 155.61 | | | |
| C | | 37.6 | 168.94 | 168.99 | | | |
| C | | 15.4 | 172.50 | 172.53 | | | |
| C | | 74.9 | 111.49 | 111.94 | | | |
| C | | 83 | 104.41 | 106.28 | | | |
| C | | 69.9 | 113.73 | 113.31 | | | |
| C | x | 173.4 | 9.53 | 10.97 | | | |
| C | x | 137.7 | 43.60 | 43.06 | | | |
| C | x | 114.9 | 68.10 | 67.30 | | | |
| C | x | 129.6 | 52.88 | 54.01 | | | |
| C | x | 124.8 | 58.18 | 58.01 | | | |
| C | x | 124.9 | 58.32 | 60.53 | | | |
| C | x | 138.3 | 44.53 | 46.25 | | | |
| C | | 31.2 | 153.514377 | 152.435037 | | | |
| C | | 18.6 | 167.966462 | 169.468824 | | | |
| C | | 17.5 | 169.390233 | 167.55976 | | | |
| | | | | | | | |
| H | | 4.79 | 27.1650146 | 26.9946404 | | | |
| H | x | 7.66 | 23.7883369 | 24.0333131 | | | |
| H | x | 7.83 | 23.613347 | 23.7976278 | | | |
| H | x | 7.53 | 23.8902455 | 24.1799099 | | | |
| H | x | 8.15 | 23.1464999 | 23.8238572 | | | |
| H | | 5.35 | 26.0592114 | 26.3940877 | | | |
| H | | 2.8 | 29.3024209 | 29.7404147 | | | |
| H | | 2.67 | 29.7417732 | 29.1010517 | | | |
| H | | 2.98 | 28.8678249 | 28.8197877 | | | |
| H | | 1.16 | 30.653933 | 30.6266757 | | | |
| H | | 0.91 | 30.8614699 | 30.9412755 | | | |
| H | | 5.01 | 26.5753067 | 25.5578008 | | | |
| H | | 3.47 | 28.5724327 | 28.5448459 | | | |
| H | x | 7.36 | 24.0726555 | 24.5656526 | | | |
| H | x | 7.33 | 24.1598288 | 25.3304038 | | | |
| H | x | 7.13 | 24.3811862 | 25.368983 | | | |
| H | x | 7.43 | 24.2264785 | 24.8494575 | | | |
| H | | 1.81 | 29.7075522 | 29.6697783 | | | |
| H | | 0.74 | 30.7708926 | 30.7243404 | | | |
| H | | 0.76 | 30.629889 | 30.72382 | | | |

| Functional | Solvent? | Basis Set | Type of Data |
|---|---|---|---|
| mPW1PW91 | PCM | 6-311G(d,p) | Shielding Tensors |

| | Isomer 1 | Isomer 2 | Isomer 3 | Isomer 4 | Isomer 5 | Isomer 6 |
|---|---|---|---|---|---|---|
| sDP4+ (H data) | 100.00% | 0.00% | - | - | - | - |
| sDP4+ (C data) | 99.97% | 0.03% | - | - | - | - |
| sDP4+ (all data) | 100.00% | 0.00% | - | - | - | - |
| uDP4+ (H data) | 100.00% | 0.00% | - | - | - | - |
| uDP4+ (C data) | 99.99% | 0.01% | - | - | - | - |
| uDP4+ (all data) | 100.00% | 0.00% | - | - | - | - |
| DP4+ (H data) | 100.00% | 0.00% | - | - | - | - |
| DP4+ (C data) | 100.00% | 0.00% | - | - | - | - |
| DP4+ (all data) | 100.00% | 0.00% | - | - | - | - |

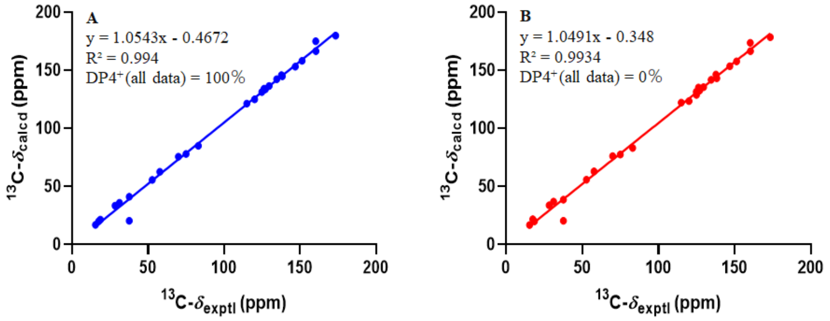


**Figure S25.** Linear regression analysis between experimental and calculated $^{13}$C NMR chemical shifts of (3*S*,14*R*,19*S*,20*R*,22*S*)-**1** (**A**) and (3*R*,14*S*,19*S*,20*R*,22*S*)-**1** (**B**).

**Table S2.** Experimental and calculated $^{13}$C NMR chemical shifts of 14*R*-**1** and 14*S*-**1**.

| No. | Experimental | Calculated ($\delta_C$, ppm) | |
|---|---|---|---|
| | ($\delta_C$, ppm) | 14*R*-**1** | 14*S*-**1** |
| 1 | 160.3 | 174.9278 | 173.6222 |
| 3 | 57.7 | 62.59371 | 62.89784 |
| 4 | 151.3 | 158.128 | 157.5464 |
| 6 | 146.8 | 153.0285 | 153.3784 |
| 7 | 127.2 | 133.9701 | 133.4517 |
| 8 | 134.6 | 142.2425 | 141.697 |
| 9 | 126.9 | 133.4278 | 132.4668 |
| 10 | 126.3 | 134.0724 | 135.0252 |
| 11 | 120.1 | 124.8501 | 123.4343 |
| 12 | 160.4 | 166.3646 | 166.4987 |
| 14 | 52.7 | 55.52872 | 55.74692 |
| 15 | 37.6 | 41.06055 | 38.66107 |
| 16 | 28.3 | 33.33352 | 33.73685 |
| 17 | 37.6 | 20.41222 | 20.3574 |
| 18 | 15.4 | 16.85284 | 16.81415 |
| 19 | 74.9 | 77.85413 | 77.41081 |
| 20 | 83 | 84.93511 | 83.06665 |
| 22 | 69.9 | 75.61515 | 76.03525 |
| 23 | 173.4 | 179.8235 | 178.3831 |
| 25 | 137.7 | 145.7528 | 146.2895 |
| 26 | 114.9 | 121.2535 | 122.0447 |
| 27 | 129.6 | 136.4737 | 135.3343 |
| 28 | 124.8 | 131.1701 | 131.3356 |
| 29 | 124.9 | 131.0293 | 128.8207 |
| 30 | 138.3 | 144.8174 | 143.0968 |
| 31 | 31.2 | 35.83442 | 36.91376 |
| 32 | 18.6 | 21.38234 | 19.87998 |
| 33 | 17.5 | 19.95857 | 21.78904 |
| **$R^2$** | | **0.994** | **0.9934** |
| **MAE** | | **6.04** | **5.65** |
| **CMAE** | | **2.07** | **2.42** |

**Table S3.** Experimental and calculated $^{1}$H NMR chemical shifts of 14*R*-**1** and 14*S*-**1**

| No. | Experimental ($\delta_H$, ppm) | Calculated ($\delta_H$, ppm) | |
|---|---|---|---|
| | | 14*R*-**1** | 14*S*-**1** |
| 3 | 4.79 | 4.725485 | 4.89586 |
| 7 | 7.66 | 8.102163 | 7.857187 |
| 8 | 7.83 | 8.277153 | 8.092872 |
| 9 | 7.53 | 8.000255 | 7.71059 |
| 10 | 8.15 | 8.744 | 8.066643 |
| 14 | 5.35 | 5.831289 | 5.496412 |
| 15a | 2.8 | 2.588079 | 2.160085 |
| 15b | 2.67 | 2.148727 | 2.789448 |
| 16 | 2.98 | 3.022675 | 3.070712 |
| 17 | 1.16 | 1.236567 | 1.263824 |
| 18 | 0.91 | 1.02903 | 0.949225 |
| 20 | 5.01 | 5.315193 | 6.332699 |
| 22 | 3.47 | 3.318067 | 3.345654 |
| 26 | 7.36 | 7.817844 | 7.324847 |
| 27 | 7.33 | 7.730671 | 6.560096 |
| 28 | 7.13 | 7.509314 | 6.521517 |
| 29 | 7.43 | 7.664021 | 7.041043 |
| 31 | 1.81 | 2.182948 | 2.220722 |
| 32 | 0.74 | 1.119607 | 1.16616 |
| 33 | 0.76 | 1.260611 | 1.16668 |
| **$R^2$** | | **0.9919** | **0.9729** |
| **MAE** | | **0.33** | **0.32** |
| **CMAE** | | **0.18** | **0.31** |

**Table S4.** Conformational analysis of the optimized isomer 14*R*-**1** and 14*S*-**1** at B3LYP/6-311G(d) level in gas phase.

| Conformations | *G* (hartree) | *ΔG* (kcal/mol) | Boltzmann distributions (%) |
|---|---|---|---|
| **14*S*-1-1** | -1660.438119 | 0 | 60.2% |
| **14*S*-1-2** | -1660.437278 | 0.527735405 | 24.7% |
| **14*S*-1-3** | -1660.436267 | 1.162147409 | 8.5% |
| **14*S*-1-4** | -1660.435525 | 1.627759383 | 3.8% |
| **14*S*-1-5** | -1660.435215 | 1.822287298 | 2.8% |
| **14*R*-1-1** | -1660.440364 | 0 | 83.8% |
| **14*R*-1-2** | -1660.438535 | 1.147714693 | 12.0% |
| **14*R*-1-3** | -1660.437175 | 2.001127477 | 2.8% |
| **14*R*-1-4** | -1660.436471 | 2.442894094 | 1.4% |

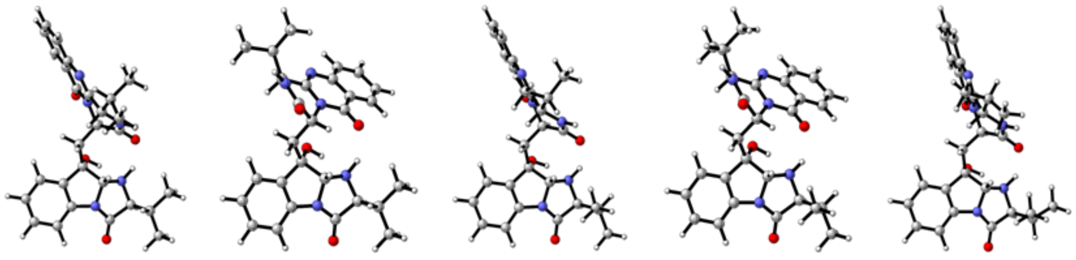

14*R*-**1**-1　14*R*-**1**-2　14*R*-**1**-3　14*R*-**1**-4　14*R*-**1**-5

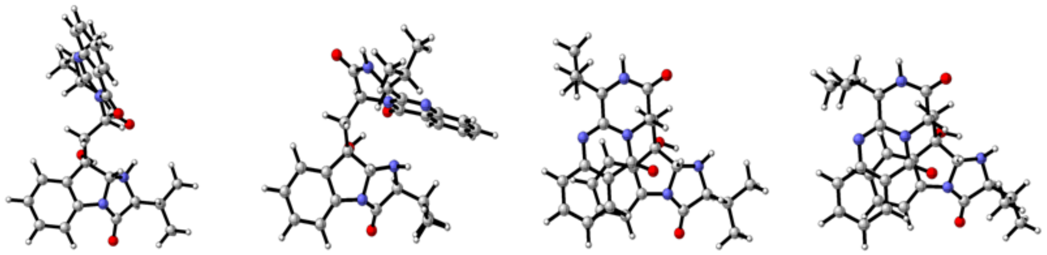

14*S*-**1**-1　14*S*-**1**-2　14*S*-**1**-3　14*S*-**1**-4

**Figure S26.** Optimized geometries of isomers 14*R*-**1** and 14*S*-**1** at B3LYP/6-311G(d) level in gas phase.

**Table S5.** The coordinates for the optimized conformers of 14*R*-**1** in the gas phase at the B3LYP/6-311G(d) level

| 14*R*-**1-1** | | | | 14*R*-**1-2** | | | | 14*R*-**1-3** | | | | 14*R*-**1-4** | | | | 14*R*-**1-5** | | | |
|---|---|---|---|---|---|---|---|---|---|---|---|---|---|---|---|---|---|---|---|
| C | 1.520291 | -3.42166 | 1.096293 | C | 1.408132 | -3.2967 | -1.56858 | C | -1.64136 | 3.234581 | 1.308402 | C | 1.512073 | -3.29023 | -1.47445 | C | 1.700149 | -3.26676 | 1.373713 |
| C | 2.344093 | -4.2693 | 1.84445 | C | 2.37315 | -4.1912 | -2.04057 | C | -2.49539 | 4.013123 | 2.097098 | C | 2.517297 | -4.13308 | -1.95795 | C | 2.5766 | -4.02613 | 2.156676 |
| C | 3.654908 | -3.89704 | 2.145892 | C | **3.727479** | -3.96867 | -1.78448 | C | -3.80654 | 3.601512 | 2.339274 | C | 3.862028 | -3.81353 | -1.76202 | C | 3.891004 | -3.604 | 2.359796 |
| C | 4.173187 | -2.67073 | 1.721137 | C | 4.1513 | -2.85637 | -1.05333 | C | -4.29565 | 2.401939 | 1.814452 | C | 4.236471 | -2.65271 | -1.08081 | C | 4.361577 | -2.41215 | 1.801378 |
| C | 3.335074 | -1.8425 | 0.987205 | C | 3.174187 | -1.98333 | -0.59157 | C | -3.42848 | 1.642243 | 1.041982 | C | 3.220445 | -1.83275 | -0.60731 | C | 3.472593 | -1.67135 | 1.035548 |
| C | 2.022281 | -2.20269 | 0.667505 | C | 1.815991 | -2.19157 | -0.83646 | C | -2.11444 | 2.044157 | 0.779076 | C | 1.870815 | -2.13757 | -0.79151 | C | 2.154413 | -2.08449 | 0.810974 |
| N | 3.627027 | -0.58167 | 0.432788 | N | 3.356015 | -0.77309 | 0.110654 | N | -3.68696 | 0.420861 | 0.388544 | N | 3.346176 | -0.58783 | 0.047368 | N | 3.707588 | -0.46133 | 0.354442 |
| C | 2.408335 | 0.03006 | -0.10375 | C | 2.05796 | -0.20591 | 0.499093 | C | -2.44239 | -0.13948 | -0.13835 | C | 2.033412 | -0.11287 | 0.497973 | C | 2.447359 | 0.083751 | -0.15047 |
| C | 1.382346 | -1.15213 | -0.23882 | C | 0.972488 | -1.05703 | -0.26062 | C | -1.43965 | 1.06894 | -0.1825 | C | 0.976993 | -1.04365 | -0.21339 | C | 1.454150 | -1.1319 | -0.15539 |
| C | 4.748962 | -0.19515 | -0.25997 | C | 4.26374 | 0.213626 | -0.21313 | C | -4.7807 | 0.081031 | -0.36833 | C | 4.159306 | 0.445938 | -0.36636 | C | 4.782803 | -0.10281 | -0.41817 |
| C | 4.287144 | 0.895526 | -1.23299 | C | 3.58176 | 1.546813 | 0.103073 | C | -4.28095 | -0.86858 | -1.46197 | C | 3.39023 | 1.744538 | -0.12417 | C | 4.246934 | 0.819995 | -1.51501 |
| N | 2.854471 | 0.573448 | -1.37557 | N | 2.158776 | 1.168365 | 0.02799 | N | -2.82282 | -0.6404 | -1.44889 | N | 2.004734 | 1.269357 | 0.040361 | N | 2.793447 | 0.570558 | -1.47489 |
| O | 5.870153 | -0.64197 | -0.11313 | O | 5.371009 | 0.035089 | -0.67955 | O | -5.91549 | 0.487124 | -0.20591 | O | 5.259899 | 0.324723 | -0.86636 | O | 5.934193 | -0.4621 | -0.2653 |
| C | 0.043735 | -0.81711 | 0.207955 | C | -0.11482 | -1.63705 | 0.648266 | C | -0.01199 | 0.731216 | 0.2584 | C | -0.04567 | -1.67267 | 0.738939 | C | 0.032347 | -0.79149 | 0.302126 |
| O | 1.365093 | -1.60672 | -1.58544 | O | 0.368364 | -0.27827 | -1.28443 | O | -1.42236 | 1.607998 | -1.49665 | O | 0.306077 | -0.32102 | -1.23548 | O | 1.419531 | -1.69506 | -1.45912 |
| C | 0.812159 | 0.156983 | -0.71061 | C | -0.99835 | -0.66568 | 1.458503 | C | 0.750654 | -0.23573 | -0.67487 | C | -0.96162 | -0.74559 | 1.566005 | C | 0.741211 | 0.1566 | -0.64126 |
| C | 0.403024 | 1.612721 | -0.54638 | C | -1.96976 | -1.50271 | 2.28151 | C | 0.392805 | -1.69952 | -0.45946 | C | -1.89267 | -1.62489 | 2.390149 | C | 0.397604 | 1.632358 | -0.46428 |
| N | 1.047945 | 2.271475 | 0.4298 | N | -3.11826 | -1.79631 | 1.635265 | N | 1.106376 | -2.30891 | 0.501313 | N | -3.03353 | -1.95249 | 1.74663 | N | 1.112107 | 2.259291 | 0.485615 |
| C | 2.055297 | 1.675713 | 1.306309 | C | -3.40065 | -1.45732 | 0.240251 | C | 2.108881 | -1.64322 | 1.337435 | C | -3.3229 | -1.63142 | 0.344866 | C | 2.066518 | 1.56791 | 1.357486 |
| C | 2.910002 | 0.700611 | 0.512261 | C | -2.84456 | -0.07853 | -0.07949 | C | 2.917324 | -0.69157 | 0.470115 | C | -2.84783 | -0.21737 | 0.04773 | C | 2.909772 | 0.662219 | 0.470018 |
| N | 2.265901 | 0.009595 | -0.49239 | N | -1.73039 | 0.308573 | 0.631913 | N | 2.208761 | -0.04712 | -0.52668 | N | -1.7437 | 0.206834 | 0.759769 | N | 2.200586 | -0.02683 | -0.49509 |
| N | 4.152166 | 0.547626 | 0.819535 | N | -3.42504 | 0.651563 | -0.96921 | N | 4.172357 | -0.5107 | 0.696523 | N | -3.47049 | 0.511666 | -0.81292 | N | 4.184582 | 0.580861 | 0.628909 |
| C | 4.899338 | -0.36186 | 0.092843 | C | -2.9106 | 1.909643 | -1.21815 | C | 4.865601 | 0.377291 | -0.10556 | C | -3.02174 | 1.799918 | -1.0296 | C | 4.897467 | -0.26523 | -0.2026 |
| C | 4.345538 | -1.09966 | -0.97222 | C | -1.83802 | 2.435101 | -0.46927 | C | 4.244018 | 1.066258 | -1.16515 | C | -1.97052 | 2.357567 | -0.27459 | C | 4.269808 | -1.02034 | -1.21501 |
| C | 2.934947 | -0.92997 | -1.30239 | C | -1.20883 | 1.602955 | 0.542156 | C | 2.820811 | 0.862973 | -1.41166 | C | -1.293 | 1.529674 | 0.708999 | C | 2.826295 | -0.90951 | -1.39654 |
| C | 6.254572 | -0.54664 | 0.418609 | C | -3.49195 | 2.701603 | -2.22422 | C | 6.234299 | 0.589809 | 0.137002 | C | -3.65176 | 2.589188 | -2.00835 | C | 6.289064 | -0.37221 | -0.03483 |
| C | 7.025385 | -1.44027 | -0.30271 | C | -3.01011 | 3.974577 | -2.46819 | C | 6.951489 | 1.46415 | -0.65943 | C | -3.23629 | 3.890944 | -2.22031 | C | 7.023326 | -1.2108 | -0.85406 |
| C | 6.469089 | -2.17204 | -1.36586 | C | -1.94303 | 4.495861 | -1.71542 | C | 6.327163 | 2.147835 | -1.71676 | C | -2.19022 | 4.444312 | -1.461 | C | 6.392771 | -1.96272 | -1.85985 |
| C | 5.137477 | -2.00308 | -1.69686 | C | -1.36242 | 3.731466 | -0.72122 | C | 4.981851 | 1.950106 | -1.96687 | C | -1.56321 | 3.682962 | -0.49294 | C | 5.024803 | -1.86734 | -2.0374 |
| O | 2.338052 | -1.53465 | -2.18139 | O | -0.2789 | 1.956963 | 1.265761 | O | 2.172471 | 1.418178 | -2.28684 | O | -0.37877 | 1.911846 | 1.438334 | O | 2.176508 | -1.50829 | -2.24150 |
| O | 0.461548 | 2.128213 | -1.25249 | O | -1.69109 | -1.89313 | 3.405625 | O | -0.48859 | -2.26191 | -1.10632 | O | -1.59379 | -2.01147 | 3.510399 | O | 0.464861 | 2.191042 | -1.13984 |
| C | 2.877351 | 2.767958 | 2.017303 | C | -4.90988 | -1.57302 | -0.05666 | C | 2.907154 | -2.72017 | 2.105646 | C | -4.82089 | -1.90913 | 0.075803 | C | 2.89311 | 2.542521 | 2.222411 |
| C | 3.574276 | 3.719388 | 1.040327 | C | -5.76206 | -0.67998 | 0.849457 | C | 3.684408 | -2.14054 | 3.290549 | C | -5.13988 | -2.0255 | -1.41705 | C | 2.045458 | 3.704008 | 2.759779 |
| C | 2.009343 | 3.528607 | 3.026087 | C | -5.36508 | -3.0355 | -0.00098 | C | 3.789855 | -3.58454 | 1.199782 | C | -5.75524 | -0.93281 | 0.796312 | C | 3.520297 | 1.784164 | 3.399204 |
| C | 4.598963 | 2.315349 | -0.69363 | C | 4.036322 | 2.144454 | 1.458428 | C | -4.70539 | -2.3435 | -1.26785 | C | 3.934615 | 2.598819 | 1.045128 | C | 4.685361 | 2.288559 | -1.32495 |
| C | 6.112057 | 2.549472 | -0.64906 | C | 5.529391 | 2.485214 | 1.426896 | C | -4.1829 | -2.94555 | 0.04002 | C | 3.941585 | 1.849011 | 2.380208 | C | 4.281151 | 3.132892 | -2.53737 |
| C | 3.907197 | 3.391214 | -1.53747 | C | 3.207611 | 3.385039 | 1.809923 | C | -6.22361 | -2.49827 | -1.39369 | C | 5.320047 | 3.158596 | 0.709643 | C | 4.155527 | 2.903033 | -0.02528 |
| H | 0.508628 | -3.7247 | 0.848818 | H | 0.35751 | -3.46197 | -1.78249 | H | -0.62864 | 3.567992 | 1.109181 | H | 0.468019 | -3.53267 | -1.64186 | H | 0.684705 | -3.60857 | 1.204844 |
| H | 1.962173 | -5.22323 | 2.190317 | H | 2.068361 | -5.06238 | -2.60945 | H | -2.13634 | 4.944031 | 2.521324 | H | 2.250888 | -5.04023 | -2.48861 | H | 2.232475 | -4.95043 | 2.606873 |

| | | | | | | | | | | | | | | | | | | | |
|---|---|---|---|---|---|---|---|---|---|---|---|---|---|---|---|---|---|---|---|
| H | 4.283398 | -4.56581 | 2.723618 | H | 4.466297 | -4.66945 | -2.15751 | H | -4.45824 | 4.217047 | 2.949522 | H | 4.632049 | -4.47549 | -2.14271 | H | 4.559981 | -4.20488 | 2.965934 |
| H | 5.188873 | -2.37484 | 1.946725 | H | 5.198933 | -2.66949 | -0.86087 | H | -5.31092 | 2.075314 | 1.994801 | H | 5.275342 | -2.39017 | -0.93502 | H | 5.378996 | -2.07698 | 1.952189 |
| H | 2.033048 | 0.78367 | 0.596746 | H | 1.925703 | -0.29261 | 1.582682 | H | -2.07104 | -0.9122 | 0.539647 | H | 1.954438 | -0.21738 | 1.583661 | H | 2.086377 | 0.862844 | 0.526711 |
| H | 4.800375 | 0.755641 | -2.18798 | H | 3.825482 | 2.2537 | -0.69482 | H | -4.70352 | -0.51578 | -2.4069 | H | 3.471178 | 2.335808 | -1.04067 | H | 4.659521 | 0.462139 | -2.46285 |
| H | 2.267568 | 1.351975 | -1.65337 | H | 1.536896 | 1.778517 | 0.546533 | H | -2.26562 | -1.45274 | -1.69109 | H | 1.434119 | 1.850346 | 0.645056 | H | 2.211887 | 1.363799 | -1.72217 |
| H | 0.612265 | -1.74917 | 0.205957 | H | -0.74801 | -2.27571 | 0.028491 | H | 0.552935 | 1.665483 | 0.264888 | H | -0.65528 | -2.35848 | 0.146895 | H | 0.531996 | -1.72559 | 0.333321 |
| H | 0.028543 | -0.46084 | 1.24088 | H | 0.361268 | -2.29704 | 1.377644 | H | -0.02434 | 0.363334 | 1.287139 | H | 0.489569 | -2.29286 | 1.462519 | H | 0.062579 | -0.40665 | 1.323753 |
| H | 1.996134 | -1.02043 | -2.05465 | H | 0.946891 | 0.506004 | -1.38086 | H | -1.96646 | 0.984846 | -2.02528 | H | 0.781199 | 0.534831 | -1.28836 | H | 1.950341 | -1.07812 | -2.00811 |
| H | 0.623211 | -0.13042 | -1.73995 | H | -0.39579 | -0.08672 | 2.150765 | H | 0.513107 | 0.026003 | -1.70165 | H | -0.37556 | -0.14892 | 2.256365 | H | 0.504819 | -0.12408 | -1.66337 |
| H | 0.769687 | 3.229393 | 0.588704 | H | -3.75175 | -2.42158 | 2.112438 | H | 0.866452 | -3.2679 | 0.712332 | H | -3.64766 | -2.60389 | 2.2157 | H | 0.889118 | 3.2273 | 0.661704 |
| H | 1.551324 | 1.08811 | 2.085792 | H | -2.88705 | -2.16911 | -0.42055 | H | 1.594024 | -1.02868 | 2.08769 | H | -2.75185 | -2.3061 | -0.30663 | H | 1.505062 | 0.92355 | 2.046787 |
| H | 6.670733 | 0.025917 | 1.239141 | H | -4.31834 | 2.29069 | -2.79202 | H | 6.704138 | 0.054652 | 0.953801 | H | -4.46182 | 2.153886 | -2.58151 | H | 6.76206 | 0.214091 | 0.744143 |
| H | 8.070088 | -1.57703 | -0.04613 | H | -3.46207 | 4.579146 | -3.2467 | H | 8.006738 | 1.623246 | -0.46686 | H | -3.72482 | 4.493369 | -2.97813 | H | 8.096387 | -1.28906 | -0.71874 |
| H | 7.084636 | -2.86801 | -1.92385 | H | -1.57773 | 5.49617 | -1.91665 | H | 6.901233 | 2.829245 | -2.33384 | H | -1.87739 | 5.466971 | -1.63678 | H | 6.97976 | -2.61612 | -2.49479 |
| H | 4.684476 | -2.55771 | -2.50948 | H | -0.5373 | 4.109692 | -0.13034 | H | 4.477175 | 2.466845 | -2.77402 | H | -0.75414 | 4.086433 | 0.103369 | H | 4.515836 | -2.43597 | -2.80601 |
| H | 3.646145 | 2.226548 | 2.570964 | H | -5.0219 | -1.22218 | -1.08386 | H | 2.127808 | -3.36729 | 2.529298 | H | -4.98213 | -2.90572 | 0.507344 | H | 3.692986 | 2.947811 | 1.596898 |
| H | 4.204702 | 4.420722 | 1.591699 | H | -6.8118 | -0.74344 | 0.553638 | H | 4.097111 | -2.95302 | 3.893887 | H | -6.16663 | -2.37647 | -1.54868 | H | 2.628418 | 4.272265 | 3.487173 |
| H | 4.210127 | 3.177497 | 0.339281 | H | -5.45866 | 0.365707 | 0.780642 | H | 3.034612 | -1.54422 | 3.93767 | H | -4.47806 | -2.74454 | -1.90865 | H | 1.744702 | 4.415492 | 1.986557 |
| H | 2.858834 | 4.311319 | 0.462986 | H | -5.6988 | -0.98344 | 1.897771 | H | 4.503758 | -1.50946 | 2.95137 | H | -5.03403 | -1.06484 | -1.91728 | H | 1.145122 | 3.345291 | 3.267622 |
| H | 2.626168 | 4.220904 | 3.603003 | H | -6.40298 | -3.11664 | -0.33106 | H | 4.178421 | -4.43578 | 1.763738 | H | -6.78665 | -1.28711 | 0.729861 | H | 4.169165 | 2.450921 | 3.97184 |
| H | 1.521331 | 2.848176 | 3.728694 | H | -4.75612 | -3.67049 | -0.64979 | H | 3.234477 | -3.98057 | 0.345683 | H | -5.50435 | -0.83957 | 1.855958 | H | 4.116454 | 0.939331 | 3.060372 |
| H | 1.229307 | 4.126192 | 2.543447 | H | -5.32224 | -3.45161 | 1.010631 | H | 4.63603 | -3.01331 | 0.818596 | H | -5.70769 | 0.059133 | 0.346496 | H | 2.743336 | 1.414979 | 4.076429 |
| H | 4.205703 | 2.37808 | 0.329044 | H | 3.867317 | 1.386345 | 2.23355 | H | -4.24192 | -2.88975 | -2.09927 | H | 3.241878 | 3.445188 | 1.134692 | H | 5.780445 | 2.260074 | -1.27707 |
| H | 6.333844 | 3.531202 | -0.22354 | H | 5.8484 | 2.886695 | 2.39181 | H | -4.49225 | -3.98986 | 0.150366 | H | 4.31156 | 2.498521 | 3.176864 | H | 4.667132 | 4.150859 | -2.44346 |
| H | 6.624804 | 1.795138 | -0.05121 | H | 6.144475 | 1.613323 | 1.20157 | H | -3.09218 | -2.92498 | 0.088463 | H | 2.941695 | 1.519247 | 2.670056 | H | 4.669324 | 2.709512 | -3.46744 |
| H | 6.536275 | 2.520365 | -1.658 | H | 5.736431 | 3.244321 | 0.665624 | H | -4.57513 | -2.41407 | 0.912437 | H | 4.592337 | 0.970083 | 2.343417 | H | 3.192962 | 3.207769 | -2.62989 |
| H | 4.194059 | 4.386208 | -1.18801 | H | 3.559782 | 3.821931 | 2.747598 | H | -6.50131 | -3.55476 | -1.36536 | H | 5.655237 | 3.840117 | 1.495252 | H | 4.550918 | 3.912906 | 0.104966 |
| H | 2.818743 | 3.330344 | -1.47836 | H | 2.146531 | 3.160407 | 1.937973 | H | -6.5911 | -2.07933 | -2.33446 | H | 5.309293 | 3.711857 | -0.23342 | H | 4.443064 | 2.320311 | 0.853576 |
| H | 4.199791 | 3.311684 | -2.58954 | H | 3.301186 | 4.148499 | 1.030726 | H | -6.7426 | -1.98955 | -0.57883 | H | 6.057987 | 2.358986 | 0.617497 | H | 3.064948 | 2.979839 | -0.04148 |

**Table S6.** The coordinates for the optimized conformers of 14*S*-**1** in the gas phase at the B3LYP/6-311G(d) level.

| 14*S*-**1-1** | | | | 14*S*-**1-2** | | | | 14*S*-**1-3** | | | | 14*S*-**1-4** | | | |
|---|---|---|---|---|---|---|---|---|---|---|---|---|---|---|---|
| C | 1.726774 | 2.656508 | 2.237212 | C | 3.14305 | -3.35773 | -0.39953 | C | -0.51908 | 0.84599 | 2.080463 | C | -0.54072 | 0.675871 | 2.078752 |
| C | 2.575505 | 3.722153 | 2.552555 | C | 4.432464 | -3.73158 | -0.00239 | C | -0.64336 | 2.231543 | 2.221097 | C | -0.69438 | 2.057414 | 2.229765 |
| C | 3.825711 | 3.834882 | 1.941605 | C | 5.244089 | -2.842 | 0.701296 | C | 0.337386 | 3.084017 | 1.716123 | C | 0.271981 | 2.934248 | 1.738973 |
| C | 4.256238 | 2.898361 | 0.998491 | C | 4.78724 | -1.56515 | 1.044744 | C | 1.46463 | 2.580317 | 1.061806 | C | 1.414244 | 2.459172 | 1.08917 |
| C | 3.394166 | 1.852309 | 0.695876 | C | 3.502705 | -1.22085 | 0.656965 | C | 1.562621 | 1.204096 | 0.925734 | C | 1.541598 | 1.086659 | 0.943106 |
| C | 2.142213 | 1.720285 | 1.302616 | C | 2.681145 | -2.09572 | -0.06632 | C | 0.582168 | 0.334683 | 1.413398 | C | 0.5755 | 0.192688 | 1.415498 |
| N | 3.611526 | 0.776521 | -0.1879 | N | 2.809315 | -0.00665 | 0.882984 | N | 2.59935 | 0.457757 | 0.326231 | N | 2.600385 | 0.366043 | 0.348217 |
| C | 2.39204 | -0.02715 | -0.32052 | C | 1.378723 | -0.25248 | 0.61977 | C | 2.189204 | -0.93883 | 0.171977 | C | 2.207214 | -1.0311 | 0.158551 |

| | | | | | | | | | | | | | | | |
|---|---|---|---|---|---|---|---|---|---|---|---|---|---|---|---|
| C | 1.448219 | 0.440522 | 0.844662 | C | 1.410103 | -1.36107 | -0.4857 | C | 1.004299 | -1.11074 | 1.187151 | C | 1.025977 | -1.24037 | 1.170428 |
| C | 4.745941 | 0.00691 | -0.31031 | C | 3.193996 | 1.223093 | 0.386595 | C | 3.948506 | 0.587814 | 0.530961 | C | 3.940589 | 0.509249 | 0.600152 |
| C | 4.264773 | -1.39591 | -0.70033 | C | 1.91364 | 1.962356 | -0.0312 | C | 4.556113 | -0.80228 | 0.315211 | C | 4.57552 | -0.87211 | 0.426437 |
| N | 2.892888 | -1.38249 | -0.16332 | N | 0.817223 | 1.031639 | 0.275285 | N | 3.388882 | -1.68789 | 0.531848 | N | 3.412432 | -1.78638 | 0.481344 |
| O | 5.887386 | 0.383023 | -0.12972 | O | 4.339365 | 1.613259 | 0.270186 | O | 4.532104 | 1.611224 | 0.833284 | O | 4.503787 | 1.540279 | 0.915964 |
| C | 0.00957 | 0.70897 | 0.409484 | C | 0.164563 | -2.25401 | -0.51217 | C | -0.09538 | -2.07148 | 0.717959 | C | -0.0548 | -2.21393 | 0.682312 |
| O | 1.434209 | -0.5341 | 1.88084 | O | 1.718066 | -0.80222 | -1.75858 | O | 1.517494 | -1.62818 | 2.412973 | O | 1.552023 | -1.76128 | 2.388684 |
| C | -0.69951 | -0.41643 | -0.37383 | C | 1.070429 | -1.73278 | -1.28327 | C | -0.80066 | -1.80824 | -0.63142 | C | -0.75912 | -1.93851 | -0.66571 |
| C | -0.5682 | -1.82093 | 0.201777 | C | 2.15047 | -2.80533 | -1.19372 | C | -1.81788 | -2.91958 | -0.85473 | C | -1.77459 | -3.04693 | -0.91128 |
| N | -1.48944 | -2.13602 | 1.150812 | N | 2.977456 | -2.66404 | -0.13687 | N | -3.05013 | -2.6451 | -0.37819 | N | -3.0089 | -2.77211 | -0.44002 |
| C | -2.50184 | -1.20443 | 1.618262 | C | 2.82011 | -1.64728 | 0.903334 | C | -3.39267 | -1.45851 | 0.404832 | C | -3.33783 | -1.6058 | 0.384852 |
| C | -3.03776 | -0.37943 | 0.456668 | C | 2.418722 | -0.32904 | 0.261428 | C | -2.66119 | -0.24413 | -0.14498 | C | -2.63011 | -0.38144 | -0.17398 |
| N | -2.12336 | -0.05578 | -0.52465 | N | 1.5984 | -0.42398 | -0.84633 | N | -1.43524 | -0.48227 | -0.72848 | N | -1.39822 | -0.61402 | -0.75509 |
| N | -4.27171 | -0.00787 | 0.447284 | N | 2.838642 | 0.782577 | 0.758533 | N | -3.19743 | 0.922672 | -0.03527 | N | -3.17229 | 0.783087 | -0.07878 |
| C | -4.71611 | 0.758274 | -0.61571 | C | 2.49041 | 1.961978 | 0.123828 | C | -2.50654 | 2.010001 | -0.53801 | C | -2.4869 | 1.871659 | -0.58495 |
| C | -3.86692 | 1.150872 | -1.67924 | C | 1.743714 | 1.966757 | -1.06934 | C | -1.2946 | 1.860827 | -1.23887 | C | -1.2696 | 1.727622 | -1.27635 |
| C | -2.46733 | 0.71788 | -1.64879 | C | 1.231987 | 0.708679 | -1.58446 | C | -0.7154 | 0.533918 | -1.38574 | C | -0.68394 | 0.403138 | -1.41564 |
| C | -6.05877 | 1.175064 | -0.64054 | C | 2.895701 | 3.186755 | 0.680979 | C | -3.03339 | 3.299503 | -0.35319 | C | -3.02459 | 3.158379 | -0.41065 |
| C | -6.53076 | 1.932164 | -1.69734 | C | 2.561182 | 4.37496 | 0.05583 | C | -2.36141 | 4.400108 | -0.85298 | C | -2.35625 | 4.261085 | -0.91066 |
| C | -5.68063 | 2.29446 | -2.7563 | C | 1.822859 | 4.373407 | -1.13992 | C | -1.15335 | 4.245752 | -1.5537 | C | -1.14167 | 4.111645 | -1.60134 |
| C | -4.35724 | 1.894929 | -2.74546 | C | 1.416054 | 3.175709 | -1.69874 | C | -0.62478 | 2.983409 | -1.74543 | C | -0.60321 | 2.852058 | -1.78346 |
| O | -1.62574 | 1.00695 | -2.48888 | O | 0.518718 | 0.605304 | -2.58332 | O | 0.319873 | 0.279534 | -1.98867 | O | 0.353946 | 0.15374 | -2.0172 |
| O | 0.291428 | -2.5997 | -0.19969 | O | 2.208389 | -3.72247 | -1.99919 | O | -1.50507 | -3.97275 | -1.39098 | O | -1.46109 | -4.09194 | -1.46267 |
| C | -3.60421 | -1.9484 | 2.395311 | C | 4.094135 | -1.53786 | 1.763708 | C | -4.92115 | -1.25945 | 0.457121 | C | -4.87582 | -1.50137 | 0.504762 |
| C | -4.29039 | -3.03627 | 1.563488 | C | 5.346378 | -1.22673 | 0.938559 | C | -5.54752 | -1.12115 | -0.93329 | C | -5.31667 | -0.60608 | 1.665967 |
| C | -3.05392 | -2.50689 | 3.712413 | C | 4.279775 | -2.79606 | 2.61946 | C | -5.58787 | -2.37447 | 1.270285 | C | -5.55982 | -1.15031 | -0.8129 |
| C | 4.383577 | -1.65216 | -2.22429 | C | 1.714956 | 3.34586 | 0.615811 | C | 5.234503 | -0.94387 | -1.06977 | C | 5.429506 | -0.99614 | -0.85761 |
| C | 3.687033 | -2.95949 | -2.61513 | C | 2.631225 | 4.38765 | -0.02974 | C | 5.737956 | -2.37517 | -1.28835 | C | 6.718302 | -0.17856 | -0.73338 |
| C | 5.854287 | -1.67259 | -2.65182 | C | 1.894528 | 3.309882 | 2.137151 | C | 6.390939 | 0.050335 | -1.21845 | C | 4.653226 | -0.62446 | -2.1249 |
| H | 0.762359 | 2.56197 | 2.724851 | H | 2.528434 | -4.04598 | -0.96947 | H | -1.28027 | 0.188409 | 2.485035 | H | -1.29128 | -0.00019 | 2.472618 |
| H | 2.261445 | 4.464767 | 3.277389 | H | 4.804915 | -4.71945 | -0.24913 | H | -1.5154 | 2.645322 | 2.71364 | H | -1.57833 | 2.448868 | 2.719406 |
| H | 4.475231 | 4.664619 | 2.198013 | H | 6.243374 | -3.1446 | 0.9943 | H | 0.219661 | 4.156675 | 1.818314 | H | 0.131106 | 4.003356 | 1.84886 |
| H | 5.225288 | 2.9744 | 0.524112 | H | 5.409306 | -0.87011 | 1.59378 | H | 2.231157 | 3.234604 | 0.669268 | H | 2.169928 | 3.132062 | 0.70723 |
| H | 1.927724 | 0.17054 | -1.29335 | H | 0.904827 | -0.65998 | 1.516033 | H | 1.853788 | -1.11083 | -0.85025 | H | 1.863976 | -1.17627 | -0.86409 |
| H | 4.869669 | -2.13346 | -0.16623 | H | 1.987092 | 2.094341 | -1.11675 | H | 5.304309 | -0.97162 | 1.094567 | H | 5.223285 | -1.04457 | 1.289958 |
| H | 2.257096 | -2.06696 | -0.55687 | H | 0.259438 | 1.382045 | 1.04268 | H | 3.455864 | -2.55148 | 0.008655 | H | 3.527574 | -2.58587 | -0.12949 |
| H | -0.5628 | 0.944832 | 1.30901 | H | 0.418262 | -3.1938 | -1.00802 | H | 0.349223 | -3.06733 | 0.644011 | H | 0.408071 | -3.20011 | 0.590794 |
| H | -0.02084 | 1.596947 | -0.22548 | H | 0.105315 | -2.51321 | 0.514374 | H | -0.8335 | -2.1312 | 1.521918 | H | -0.79195 | -2.3023 | 1.484009 |
| H | 2.109092 | -1.19309 | 1.61812 | H | 0.971184 | -0.25695 | -2.07219 | H | 2.433591 | -1.9045 | 2.199187 | H | 2.443268 | -2.09084 | 2.148588 |
| H | -0.29643 | -0.46215 | -1.38133 | H | 0.81592 | -1.62065 | -2.3323 | H | -0.08822 | -1.86255 | -1.4477 | H | -0.04297 | -1.98206 | -1.47941 |
| H | -1.41647 | -3.04992 | 1.548863 | H | 3.652359 | -3.40104 | 0.007948 | H | -3.72488 | -3.39509 | -0.42785 | H | -3.69367 | -3.51307 | -0.50257 |
| H | -2.03029 | -0.49974 | 2.316484 | H | 2.000362 | -1.9375 | 1.57515 | H | -3.05011 | -1.59577 | 1.44026 | H | -2.95028 | -1.76497 | 1.400514 |
| H | -6.70328 | 0.887482 | 0.181736 | H | 3.467803 | 3.172762 | 1.601114 | H | -3.96602 | 3.403683 | 0.188635 | H | -3.96186 | 3.259798 | 0.12364 |
| H | -7.56769 | 2.249257 | -1.70943 | H | 2.872183 | 5.317314 | 0.49278 | H | -2.7708 | 5.392937 | -0.70203 | H | -2.77345 | 5.251862 | -0.76781 |

| | | | | | | | | | | | | | | | |
|---|---|---|---|---|---|---|---|---|---|---|---|---|---|---|---|
| H | -6.06377 | 2.887129 | -3.57884 | H | 1.56651 | 5.311551 | -1.618 | H | -0.635 | 5.117176 | -1.9366 | H | -0.62641 | 4.984827 | -1.98443 |
| H | -3.68133 | 2.164487 | -3.54775 | H | 0.828945 | 3.148766 | -2.60811 | H | 0.312027 | 2.838159 | -2.26862 | H | 0.337849 | 2.710549 | -2.29999 |
| H | -4.34669 | -1.18481 | 2.633396 | H | 3.911202 | -0.69619 | 2.434078 | H | -5.06452 | -0.31848 | 0.990748 | H | -5.1864 | -2.52226 | 0.762824 |
| H | -5.11397 | -3.47386 | 2.132384 | H | 6.202017 | -1.08479 | 1.602777 | H | -6.61665 | -0.91617 | -0.84113 | H | -6.39642 | -0.69435 | 1.810853 |
| H | -4.70048 | -2.63243 | 0.637182 | H | 5.22324 | -0.31541 | 0.352304 | H | -5.09727 | -0.30150 | -1.4946 | H | -4.83153 | -0.90183 | 2.600837 |
| H | -3.60633 | -3.84951 | 1.306136 | H | 5.59789 | -2.04042 | 0.253066 | H | -5.43909 | -2.03555 | -1.52245 | H | -5.07619 | 0.437061 | 1.470494 |
| H | -3.86454 | -2.93672 | 4.304791 | H | 5.116925 | -2.65997 | 3.307461 | H | -6.64971 | -2.15638 | 1.403099 | H | -6.64092 | -1.25435 | -0.72137 |
| H | -2.57759 | -1.7262 | 4.311111 | H | 3.388388 | -3.01212 | 3.21413 | H | -5.13865 | -2.47166 | 2.262099 | H | -5.21954 | -1.75448 | -1.63926 |
| H | -2.31854 | -3.30237 | 3.555571 | H | 4.504329 | -3.68339 | 2.019082 | H | -5.52555 | -3.34995 | 0.777265 | H | -5.36238 | -0.08532 | -1.07317 |
| H | 3.880674 | -0.82711 | -2.74439 | H | 0.678022 | 3.625735 | 0.393876 | H | 4.478912 | -0.72022 | -1.83352 | H | 5.708283 | -2.05559 | -0.92422 |
| H | 3.816355 | -3.15349 | -3.68268 | H | 2.454808 | 5.375237 | 0.403775 | H | 6.239348 | -2.45564 | -2.25568 | H | 7.353092 | -0.33321 | -1.6092 |
| H | 2.611976 | -2.93478 | -2.42172 | H | 2.451973 | 4.455734 | -1.10629 | H | 4.934518 | -3.1162 | -1.28706 | H | 7.290187 | -0.46855 | 0.152241 |
| H | 4.111292 | -3.80761 | -2.06814 | H | 3.681335 | 4.130415 | 0.120844 | H | 6.458283 | -2.65669 | -0.51392 | H | 6.501174 | 0.888632 | -0.65574 |
| H | 5.936052 | -1.81631 | -3.73194 | H | 1.628715 | 4.274498 | 2.575295 | H | 6.854596 | -0.05497 | -2.20222 | H | 5.273495 | -0.78713 | -3.00919 |
| H | 6.368154 | -0.7463 | -2.39199 | H | 1.267894 | 2.552498 | 2.616127 | H | 6.062203 | 1.083259 | -1.10528 | H | 3.748661 | -1.22405 | -2.24868 |
| H | 6.386226 | -2.49638 | -2.16489 | H | 2.933916 | 3.09824 | 2.40346 | H | 7.162402 | -0.13802 | -0.46491 | H | 4.35854 | 0.428905 | -2.11783 |

**Table S7.** The DP4+ analysis results of compound **2**

| Functional | | Solvent? | | Basis Set | | Type of Data | |
|---|---|---|---|---|---|---|---|
| mPW1PW91 | | PCM | | 6-311G(d,p) | | Shielding Tensors | |
| | | DP4+ | 0.00% | 100.00% | - | - | - |
| Nuclei | sp2? | xperimenta | Isomer 1 | Isomer 2 | Isomer 3 | Isomer 4 | Isomer 5 |
| C | x | 161.2 | 22.3 | 22.0 | | | |
| C | x | 156.6 | 23.7 | 24.4 | | | |
| C | x | 146.2 | 38.0 | 37.4 | | | |
| C | x | 127.3 | 56.2 | 55.7 | | | |
| C | x | 135.3 | 46.5 | 46.7 | | | |
| C | x | 127.5 | 55.7 | 55.6 | | | |
| C | x | 126.2 | 55.2 | 55.4 | | | |
| C | x | 120.1 | 64.5 | 64.5 | | | |
| C | | 80.2 | 112.5 | 107.2 | | | |
| C | | 32.6 | 151.2 | 151.3 | | | |
| C | | 54.8 | 130.5 | 130.1 | | | |
| C | | 30.8 | 156.23 | 155.68 | | | |
| C | | 86.1 | 96.39 | 96.81 | | | |
| C | | 85.1 | 97.85 | 97.72 | | | |
| C | | 59.5 | 124.16 | 124.12 | | | |
| C | x | 177.2 | 5.28 | 5.30 | | | |
| C | x | 140.9 | 39.42 | 39.55 | | | |
| C | x | 117.1 | 65.54 | 65.67 | | | |
| C | x | 131.5 | 50.20 | 50.31 | | | |
| C | x | 126 | 56.74 | 56.79 | | | |
| C | x | 125.5 | 57.04 | 56.97 | | | |
| C | x | 132.3 | 52.66 | 52.30 | | | |
| C | x | 171.1 | 10.33 | 10.37 | | | |
| C | | 17.6 | 170.06 | 169.99 | | | |
| C | | 19.2 | 167.32 | 171.00 | | | |
| C | | 19.7 | 174.202099 | 168.227562 | | | |
| | | | | | | | |
| H | x | 7.72 | 23.7712484 | 23.7899131 | | | |
| H | x | 7.89 | 23.5854925 | 23.6018727 | | | |
| H | x | 7.59 | 23.8813497 | 23.8886834 | | | |
| H | x | 8.19 | 23.1675796 | 23.174623 | | | |
| H | | 4.35 | 27.1164889 | 27.1780259 | | | |
| H | | 2.14 | 29.7902215 | 29.6911649 | | | |
| H | | 6.07 | 26.3972711 | 25.8279235 | | | |
| H | | 2.76 | 29.4808396 | 29.474915 | | | |
| H | | 3.41 | 28.2189441 | 28.1516821 | | | |
| H | | 5.44 | 26.5583457 | 26.5583053 | | | |
| H | | 3.61 | 28.0599853 | 28.0589772 | | | |
| H | x | 7.45 | 24.0228135 | 24.0281881 | | | |
| H | x | 7.55 | 23.8890379 | 23.89918 | | | |
| H | x | 7.38 | 24.0854013 | 24.0874716 | | | |
| H | x | 7.67 | 23.8167522 | 23.7783208 | | | |
| H | | 1.36 | 30.4468355 | 30.44513 | | | |
| H | | 0.83 | 31.1989853 | 30.8467772 | | | |
| H | | 1.14 | 30.6373442 | 30.9007946 | | | |

| Functional | Solvent? | | Basis Set | | Type of Data | |
|---|---|---|---|---|---|---|
| mPW1PW91 | PCM | | 6-311G(d,p) | | Shielding Tensors | |
| | Isomer 1 | Isomer 2 | Isomer 3 | Isomer 4 | Isomer 5 | Isomer 6 |
| sDP4+ (H data) | 1.37% | 98.63% | - | - | - | - |
| sDP4+ (C data) | 0.00% | 100.00% | - | - | - | - |
| sDP4+ (all data) | 0.00% | 100.00% | - | - | - | - |
| uDP4+ (H data) | 4.81% | 95.19% | - | - | - | - |
| uDP4+ (C data) | 0.03% | 99.97% | - | - | - | - |
| uDP4+ (all data) | 0.00% | 100.00% | - | - | - | - |
| DP4+ (H data) | 0.07% | 99.93% | - | - | - | - |
| DP4+ (C data) | 0.00% | 100.00% | - | - | - | - |
| DP4+ (all data) | 0.00% | 100.00% | - | - | - | - |

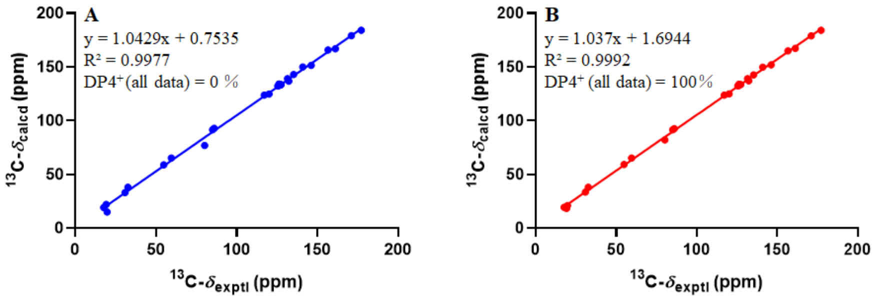


**Figure 27.** Linear regression analysis between experimental and calculated $^{13}$C NMR chemical shifts of (11*S*,13*S*,14*R*,16*S*,27*R*)-**2** (**A**) and (11*S*,13*S*,14*R*,16*S*,27*S*)-**2** (**B**).

**Table S8.** Experimental and calculated $^{13}$C NMR chemical shifts of 27*R*-**2** and 27*S*-**2**.

| No. | Experimental ($\delta_C$, ppm) | Calculated ($\delta_C$, ppm) 27*R*-**2** | Calculated ($\delta_C$, ppm) 27*S*-**2** |
|---|---|---|---|
| 1 | 161.2 | 167.0 | 167.3 |
| 3 | 156.6 | 165.7 | 164.9 |
| 5 | 146.2 | 151.4 | 152.0 |
| 6 | 127.3 | 133.2 | 133.7 |
| 7 | 135.3 | 142.9 | 142.7 |
| 8 | 127.5 | 133.6 | 133.7 |
| 9 | 126.2 | 134.1 | 134.0 |
| 10 | 120.1 | 124.8 | 124.9 |
| 11 | 54.8 | 58.9 | 59.3 |
| 12 | 30.8 | 33.1 | 33.7 |
| 13 | 86.1 | 93.0 | 92.5 |
| 14 | 85.1 | 91.5 | 91.6 |
| 16 | 59.5 | 65.2 | 65.2 |
| 17 | 177.2 | 184.1 | 184.0 |
| 19 | 140.9 | 149.9 | 149.8 |
| 20 | 117.1 | 123.8 | 123.7 |
| 21 | 131.5 | 139.1 | 139.0 |
| 22 | 126 | 132.6 | 132.6 |
| 23 | 150.5 | 132.3 | 132.4 |
| 24 | 132.3 | 136.7 | 137.0 |
| 26 | 171.1 | 179.0 | 179.0 |
| 27 | 80.2 | 76.8 | 82.1 |
| 28 | 32.6 | 38.1 | 38.0 |
| 29 | 19.7 | 15.1 | 21.1 |

| | | | |
|---|---|---|---|
| 30 | 19.2 | 22.0 | 18.3 |
| 31 | 17.6 | 19.3 | 19.4 |
| **$R^2$** | | **0.9977** | **0.9992** |
| **MAE** | | **5.83** | **5.62** |
| **CMAE** | | **1.68** | **1.15** |

**Table S9.** Experimental and calculated $^1$H NMR chemical shifts of 27*R*-**2** and 27*S*-**2**.

| No. | Experimental ($\delta_H$, ppm) | Calculated ($\delta_H$, ppm) | |
|---|---|---|---|
| | | 27*R*-**2** | 27*S*-**2** |
| 6 | 7.72 | 8.12 | 8.10 |
| 7 | 7.89 | 8.31 | 8.29 |
| 8 | 7.59 | 8.01 | 8.00 |
| 9 | 8.19 | 8.72 | 8.72 |
| 11 | 6.07 | 5.49 | 6.06 |
| 12a | 2.76 | 2.41 | 2.42 |
| 12b | 3.41 | 3.67 | 3.74 |
| 14 | 5.44 | 5.33 | 5.33 |
| 16 | 3.61 | 3.83 | 3.83 |
| 20 | 7.45 | 7.87 | 7.86 |
| 21 | 7.55 | 8.00 | 7.99 |
| 22 | 7.38 | 7.81 | 7.80 |
| 23 | 7.67 | 8.07 | 8.11 |
| 27 | 4.35 | 4.77 | 4.71 |
| 28 | 2.14 | 2.10 | 2.20 |
| 29 | 1.14 | 1.25 | 0.99 |
| 30 | 0.83 | 0.69 | 1.04 |
| 31 | 1.36 | 1.44 | 1.45 |
| **$R^2$** | | **0.9916** | **0.9957** |
| **MAE** | | **0.32** | **0.30** |
| **CMAE** | | **0.17** | **0.13** |

**Table S10.** Conformational analysis of the optimized isomer 27*R*-**2** and 27*S*-**2** at B3LYP/6-311G(d) level in gas phase.

| Conformations | *G* (hartree) | *ΔG* (kcal/mol) | Boltzmann distributions (%) |
|---|---|---|---|
| **27*S*-2-1** | -1601.704473 | 1.986067 | 1.8 |
| **27*S*-2-2** | -1601.707493 | 0.090989 | 43.6 |
| **27*S*-2-3** | -1601.705181 | 1.541791 | 3.7 |
| **27*S*-2-4** | -1601.707638 | 0 | 50.9 |
| **27*R*-2-1** | -1601.709344 | 0 | 95.1 |
| **27*R*-2-2** | -1601.706231 | 1.953437 | 3.5 |
| **27*R*-2-3** | -1601.705366 | 2.496232 | 1.4 |

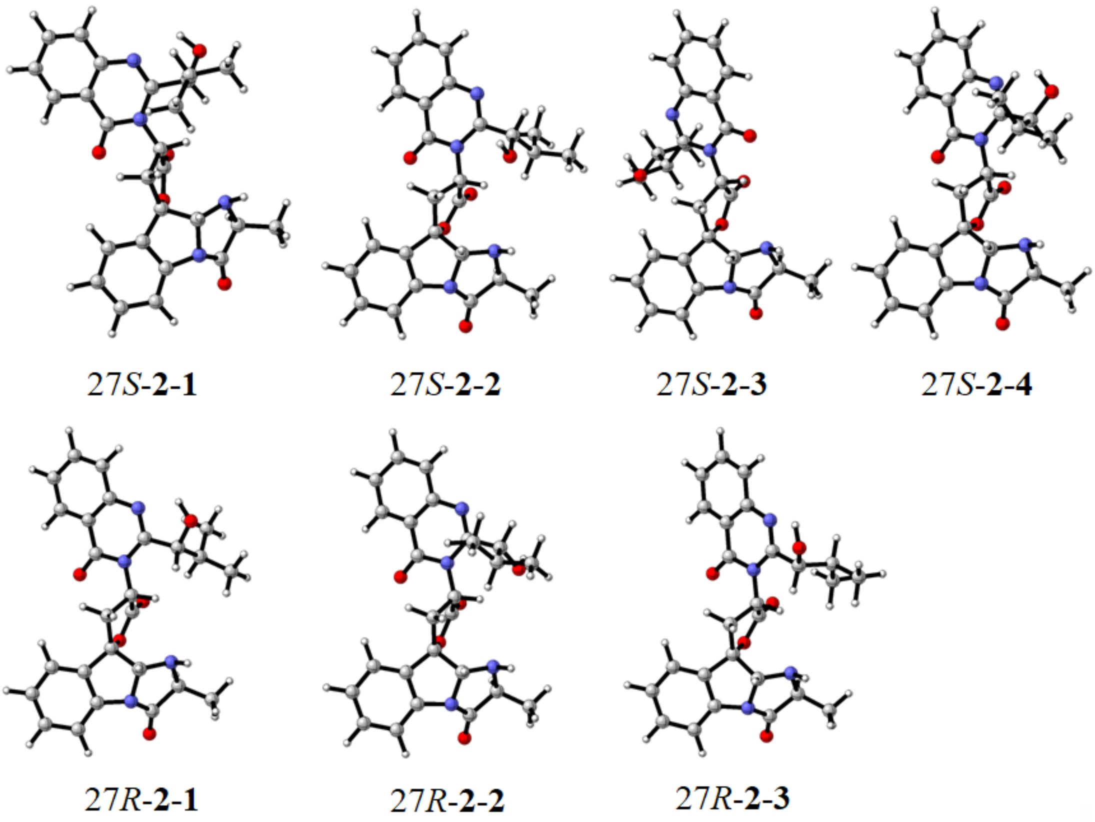


**Figure S28.** Optimized geometries of isomers 27*R*-**2** and 27*S*-**2** at B3LYP/6-311G(d) level in gas phase.

**Table S11.** The coordinates for the optimized conformers of 27*S*-**2** in the gas phase at the B3LYP/6-311G(d) level.

| 27*S*-**2-1** | | | | 27*S*-**2-2** | | | | 27*S*-**2-3** | | | | 27*S*-**2-4** | | | |
|---|---|---|---|---|---|---|---|---|---|---|---|---|---|---|---|
| C | 3.613558 | -3.65557 | 1.311273 | C | 3.693688 | -3.96647 | 0.441341 | C | 3.346861 | 3.24166 | -1.33427 | C | 3.575439 | -3.58954 | 1.55534 |

| | | | | | | | | | | | | | | | |
|---|---|---|---|---|---|---|---|---|---|---|---|---|---|---|---|
| C | 4.918952 | -3.17568 | 1.193392 | C | 4.978548 | -3.42465 | 0.380596 | C | 4.675751 | 2.830306 | -1.21893 | C | 4.884423 | -3.12709 | 1.410699 |
| C | 5.176252 | -1.83659 | 0.881765 | C | 5.185435 | -2.04137 | 0.390955 | C | 5.005921 | 1.490951 | -0.98625 | C | 5.151912 | -1.81502 | 1.006572 |
| C | 4.084426 | -1.00513 | 0.694109 | C | 4.066009 | -1.23008 | 0.462473 | C | 3.961137 | 0.588834 | -0.87105 | C | 4.066467 | -0.99209 | 0.754945 |
| C | 2.767682 | -1.47722 | 0.797954 | C | 2.767481 | -1.76096 | 0.510502 | C | 2.621663 | 0.992952 | -0.96823 | C | 2.746107 | -1.448 | 0.884329 |
| C | 2.522539 | -2.80553 | 1.104254 | C | 2.572633 | -3.13224 | 0.498642 | C | 2.30182 | 2.321118 | -1.20209 | C | 2.490967 | -2.74985 | 1.282373 |
| N | 4.069227 | 0.372923 | 0.360481 | N | 3.999102 | 0.186083 | 0.47558 | N | 4.018414 | -0.80644 | -0.62581 | N | 4.061498 | 0.359337 | 0.326306 |
| C | 2.708082 | 0.884457 | 0.606745 | C | 2.641893 | 0.574668 | 0.901476 | C | 2.690008 | -1.37751 | -0.91985 | C | 2.702233 | 0.894059 | 0.526234 |
| C | 1.819011 | -0.37502 | 0.406619 | C | 1.772978 | -0.63235 | 0.444951 | C | 1.733804 | -0.18284 | -0.6575 | C | 1.807471 | -0.37124 | 0.407286 |
| C | 4.584081 | 0.901459 | 0.808635 | C | 4.442162 | 1.000438 | -0.54952 | C | 4.557362 | -1.38217 | 0.50911 | C | 4.591662 | 0.807964 | -0.86882 |
| C | 3.580105 | 1.9454 | 1.316089 | C | 3.392495 | 2.102909 | -0.74039 | C | 3.611854 | -2.51154 | 0.938821 | C | 3.598904 | 1.821619 | -1.45411 |
| N | 2.50269 | 1.987636 | 0.306019 | N | 2.363421 | 1.85629 | 0.289453 | N | 2.537265 | -2.54017 | -0.07426 | N | 2.510312 | 1.933473 | -0.46134 |
| C | 0.435791 | -0.27921 | 1.046445 | C | 0.420437 | -0.73785 | 1.144177 | C | 0.355228 | -0.29585 | -1.3082 | C | 0.417726 | -0.22122 | 1.020765 |
| C | -0.39032 | 0.352784 | 0.077608 | C | -0.43297 | 0.188886 | 0.275394 | C | -0.48321 | -0.94732 | -0.20617 | C | -0.3865 | 0.342899 | -0.1529 |
| C | 0.343992 | -0.06183 | 1.356927 | C | 0.216054 | 0.11008 | -1.10177 | C | 0.2632 | -0.65094 | 1.088895 | C | 0.352238 | -0.17496 | -1.3915 |
| O | 1.57121 | -0.5101 | 1.03678 | O | 1.448945 | -0.42053 | -0.97634 | O | 1.480058 | -0.15285 | 0.796685 | O | 1.572118 | -0.60927 | -1.02567 |
| N | -1.80737 | -0.00166 | 0.132311 | N | -1.8664 | -0.0836 | 0.234541 | N | -1.90114 | -0.59186 | -0.1625 | N | -1.80842 | 0.010128 | -0.18687 |
| C | -2.07677 | -1.37054 | 0.363698 | C | -2.2362 | -1.32923 | -0.30797 | C | -2.80032 | -1.65542 | -0.42321 | C | -2.11173 | -1.35598 | -0.37444 |
| C | -3.48636 | -1.72494 | 0.391451 | C | -3.66343 | -1.60955 | -0.27629 | C | -4.20463 | -1.26903 | -0.46029 | C | -3.532 | -1.66739 | -0.43155 |
| C | -4.44996 | -0.70649 | 0.266159 | C | -4.5381 | -0.63434 | 0.239134 | C | -4.55449 | 0.077184 | -0.24811 | C | -4.46795 | -0.61976 | -0.34172 |
| N | -4.08094 | 0.614643 | 0.122091 | N | -4.08162 | 0.588502 | 0.69415 | N | -3.60143 | 1.038759 | 0.021474 | N | -4.0619 | 0.691464 | -0.18875 |
| C | -2.82915 | 0.929058 | 0.06302 | C | -2.81218 | 0.834663 | 0.667565 | C | -2.35672 | 0.7007 | 0.066268 | C | -2.80297 | 0.966699 | -0.11455 |
| C | -3.8817 | -3.06168 | 0.553493 | C | -4.16044 | -2.83314 | -0.74987 | C | -5.19597 | -2.22778 | -0.71862 | C | -3.96368 | -2.99301 | -0.5886 |
| C | -5.2261 | -3.38121 | 0.581885 | C | -5.51859 | -3.08704 | -0.70391 | C | -6.52432 | -1.84756 | -0.7685 | C | -5.31626 | -3.27203 | -0.65122 |
| C | -6.19152 | -2.36706 | 0.45488 | C | -6.39636 | -2.11851 | -0.18653 | C | -6.87788 | -0.50323 | -0.56085 | C | -6.25313 | -2.22837 | -0.5608 |
| C | -5.81348 | -1.04559 | 0.301449 | C | -5.91693 | -0.9074 | 0.278436 | C | -5.90892 | 0.449536 | -0.3033 | C | -5.83911 | -0.91703 | -0.40905 |
| C | -2.57311 | 2.428485 | 0.104915 | C | -2.40378 | 2.243686 | 1.084947 | C | -1.37299 | 1.844876 | 0.302508 | C | -2.45748 | 2.435263 | 0.110872 |
| C | -2.72076 | 2.854166 | 1.585341 | C | -1.94232 | 3.131765 | -0.08739 | C | -1.81088 | 2.780179 | 1.439352 | C | -1.9743 | 2.697005 | 1.563609 |
| O | -0.05627 | 0.020541 | 2.484289 | O | -0.23246 | 0.486215 | -2.15048 | O | -0.12975 | -0.80551 | 2.215079 | O | -0.03379 | -0.16759 | -2.5266 |
| O | 5.610408 | 0.557891 | 1.356208 | O | 5.44652 | 0.835176 | -1.20943 | O | 5.561002 | -1.01929 | 1.085713 | O | 5.622309 | 0.425483 | -1.38150 |
| C | 4.206614 | 3.30383 | 1.592478 | C | 3.975175 | 3.507594 | -0.69455 | C | 4.312223 | -3.86008 | 1.11978 | C | 4.236355 | 3.156428 | -1.80925 |
| O | -1.14883 | -2.15359 | 0.520412 | O | -1.37849 | -2.08228 | -0.75323 | O | -2.38935 | -2.79179 | -0.61052 | O | -1.20435 | -2.17073 | -0.47868 |
| O | -3.49828 | 3.124095 | 0.716628 | O | -1.37977 | 2.260713 | 2.08169 | O | -1.20285 | 2.532564 | -0.94264 | O | -3.58713 | 3.227803 | -0.19296 |
| C | -1.7995 | 2.080071 | 2.530466 | C | -2.87334 | 2.999685 | -1.29545 | C | -1.94053 | 2.015582 | 2.760709 | C | -1.50138 | 4.146792 | 1.686159 |
| C | -2.49509 | 4.3615 | 1.715929 | C | -1.83897 | 4.584048 | 0.386147 | C | -0.81809 | 3.938328 | 1.568556 | C | -3.06478 | 2.367618 | 2.584651 |
| H | 3.444424 | -4.69682 | 1.560232 | H | 3.563731 | -5.04253 | 0.439332 | H | 3.122548 | 4.285057 | -1.5239 | H | 3.39839 | -4.60943 | 1.876654 |
| H | 5.752527 | -3.85106 | 1.350572 | H | 5.835764 | -4.08666 | 0.329521 | H | 5.471265 | 3.560548 | -1.31732 | H | 5.71285 | -3.79469 | 1.619728 |
| H | 6.186769 | -1.4608 | 0.789038 | H | 6.17988 | -1.61726 | 0.34451 | H | 6.035058 | 1.168339 | -0.89837 | H | 6.165364 | -1.45305 | 0.893424 |
| H | 1.508183 | -3.18391 | 1.165589 | H | 1.573187 | -3.55245 | 0.51779 | H | 1.268098 | 2.64349 | -1.26943 | H | 1.474057 | -3.11727 | 1.364908 |
| H | 2.61379 | 1.205671 | 1.646291 | H | 2.596287 | 0.635393 | 1.990974 | H | 2.623085 | -1.6403 | -1.97777 | H | 2.602681 | 1.285968 | 1.540683 |
| H | 3.188197 | 1.53043 | 2.251773 | H | 2.966862 | 1.920855 | -1.734 | H | 3.200424 | -2.18606 | 1.901339 | H | 3.215566 | 1.349621 | -2.36603 |
| H | 2.537157 | 2.861638 | 0.203413 | H | 2.385735 | 2.584188 | 0.992284 | H | 2.604395 | -3.38121 | -0.63303 | H | 2.539127 | 2.840018 | -0.01189 |
| H | 0.056097 | -1.26514 | 1.297855 | H | 0.049096 | -1.7588 | 1.117146 | H | -0.03554 | 0.682251 | -1.58181 | H | 0.022009 | -1.18592 | 1.326136 |
| H | 0.457616 | 0.327111 | 1.949162 | H | 0.471483 | -0.40351 | 2.179609 | H | 0.390956 | -0.90495 | -2.21023 | H | 0.431566 | 0.43992 | 1.885908 |
| H | -0.30029 | 1.432596 | 0.032104 | H | -0.28891 | 1.201283 | 0.630509 | H | -0.46912 | -2.03168 | -0.31464 | H | -0.27726 | 1.42224 | -0.19498 |

| | | | | | | | | | | | | | | | |
|---|---|---|---|---|---|---|---|---|---|---|---|---|---|---|---|
| H | -3.11888 | -3.8241 | 0.652452 | H | -3.46372 | -3.56297 | -1.1437 | H | -4.89874 | -3.25699 | -0.87735 | H | -3.22213 | -3.77921 | -0.65889 |
| H | -5.53811 | -4.4119 | 0.703306 | H | -5.90848 | -4.03108 | -1.0664 | H | -7.29393 | -2.58421 | -0.9675 | H | -5.6559 | -4.29411 | -0.77101 |
| H | -7.24467 | -2.624 | 0.479942 | H | -7.46085 | -2.32269 | -0.15404 | H | -7.92150 | -0.21072 | -0.60173 | H | -7.31507 | -2.45354 | -0.61143 |
| H | -6.54722 | -0.25391 | 0.206631 | H | -6.58189 | -0.14922 | 0.674933 | H | -6.166 | 1.489139 | -0.1381 | H | -6.55119 | -0.10328 | -0.33923 |
| H | -1.5896 | 2.718531 | 0.263988 | H | -3.32547 | 2.669044 | 1.490523 | H | -0.37803 | 1.483568 | 0.542974 | H | -1.66729 | 2.733514 | -0.5855 |
| H | -3.75997 | 2.627238 | 1.852484 | H | -0.94413 | 2.804325 | -0.39388 | H | -2.79385 | 3.177702 | 1.168603 | H | -1.11773 | 2.045789 | 1.760735 |
| H | 3.462592 | 3.996311 | 1.991121 | H | 3.198469 | 4.252187 | -0.87993 | H | 3.608072 | -4.60843 | 1.467829 | H | 3.501541 | 3.825286 | -2.26164 |
| H | 5.006056 | 3.199701 | 2.327517 | H | 4.742405 | 3.614669 | -1.4628 | H | 5.106715 | -3.75448 | 1.861377 | H | 5.044728 | 3.001099 | -2.52522 |
| H | 4.630646 | 3.732736 | 0.680451 | H | 4.430359 | 3.711664 | 0.278387 | H | 4.756227 | -4.1901 | 0.180291 | H | 4.650692 | 3.641 | -0.92104 |
| H | -4.33595 | 2.641041 | 0.635462 | H | -1.7315 | 1.886965 | 2.898497 | H | -2.07107 | 2.852172 | -1.22433 | H | -4.34917 | 2.622044 | -0.18342 |
| H | -1.97331 | 2.392408 | 3.562406 | H | -2.56978 | 3.699323 | -2.07741 | H | -2.25189 | 2.693805 | 3.558704 | H | -1.14762 | 4.353307 | 2.699181 |
| H | -1.96402 | 1.000931 | 2.48734 | H | -2.8427 | 1.995794 | -1.72312 | H | -2.67956 | 1.214299 | 2.698447 | H | -0.68086 | 4.357693 | 0.994043 |
| H | -0.74682 | 2.278112 | 2.308351 | H | -3.90924 | 3.224576 | -1.02633 | H | -0.98755 | 1.567635 | 3.05331 | H | -2.31787 | 4.836685 | 1.463043 |
| H | -2.69502 | 4.692973 | 2.738026 | H | -1.432 | 5.215958 | -0.40698 | H | -1.14235 | 4.631993 | 2.348173 | H | -2.71278 | 2.580338 | 3.596631 |
| H | -3.1471 | 4.915797 | 1.040936 | H | -1.19066 | 4.670391 | 1.258822 | H | -0.72748 | 4.494016 | 0.634678 | H | -3.35696 | 1.315454 | 2.548208 |
| H | -1.45789 | 4.621372 | 1.478556 | H | -2.82573 | 4.975878 | 0.653345 | H | 0.177459 | 3.572406 | 1.841295 | H | -3.95679 | 2.971458 | 2.404761 |

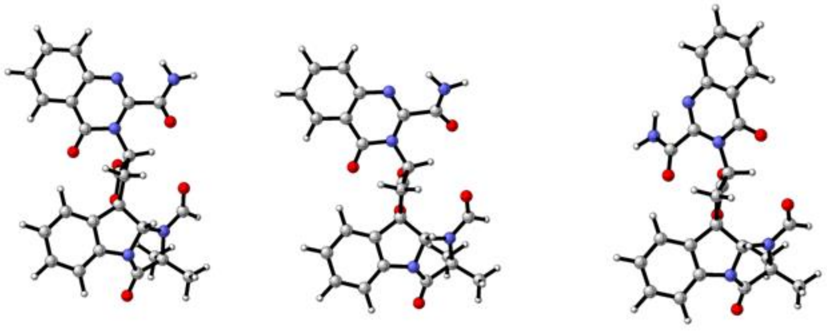

3-1 3-2 3-3

**Figure S29.** Optimized geometries of isomers **3** at B3LYP/6-311G(d) level in gas phase.

**Table S12.** The coordinates for the optimized conformers of **3** in the gas phase at the B3LYP/6-311G(d) level.

| **34-1** | | | | **34-2** | | | | **34-3** | | | |
|---|---|---|---|---|---|---|---|---|---|---|---|
| C | -1.77216 | 3.58229 | -1.08039 | C | -2.33226 | 3.27225 | -0.77704 | C | -0.60065 | 4.72826 | 0.46151 |
| C | -2.57913 | 3.69736 | -2.21908 | C | -3.54432 | 3.16465 | -1.47131 | C | -0.07961 | 5.89666 | 1.03097 |
| C | -3.16039 | 4.90889 | -2.52996 | C | -4.36544 | 4.2656 | -1.59229 | C | -0.65358 | 7.1176 | 0.74389 |
| C | -2.94856 | 6.02178 | -1.70927 | C | -3.99183 | 5.48849 | -1.02349 | C | -1.7543 | 7.1921 | -0.11582 |
| C | -2.16471 | 5.92359 | -0.57869 | C | -2.80035 | 5.6124 | -0.34105 | C | -2.28279 | 6.05081 | -0.68189 |
| C | -1.56694 | 4.69789 | -0.25326 | C | -1.95617 | 4.49934 | -0.21364 | C | -1.70783 | 4.80452 | -0.39626 |
| N | -0.82419 | 4.59001 | 0.89845 | N | -0.76405 | 4.62495 | 0.45245 | N | -2.25988 | 3.665 | -0.93224 |
| C | -0.2257 | 3.47178 | 1.15704 | C | 0.02379 | 3.60208 | 0.57034 | C | -1.70189 | 2.52357 | -0.69094 |
| N | -0.25941 | 2.3823 | 0.3355 | N | -0.28342 | 2.35735 | 0.09335 | N | -0.55864 | 2.36334 | 0.03941 |
| C | -1.12455 | 2.31926 | -0.76415 | C | -1.46954 | 2.1154 | -0.61717 | C | 0.02988 | 3.44257 | 0.72516 |

| | | | | | | | | | | | |
|---|---|---|---|---|---|---|---|---|---|---|---|
| C | 0.587 | 1.21336 | 0.5499 | C | 0.55964 | 1.17745 | 0.27469 | C | 0.21668 | 1.12803 | 0.07464 |
| C | 1.13134 | 0.67972 | -0.78845 | C | 0.84815 | 0.60094 | -1.07515 | C | 0.09953 | 0.27466 | -1.18627 |
| O | 0.95562 | -0.6463 | -0.83247 | O | 0.70446 | -0.82338 | -0.93334 | O | 0.15009 | -1.01569 | -0.83446 |
| C | 0.27078 | -1.18059 | 0.29997 | C | 0.21549 | -1.22359 | 0.34728 | C | 0.17174 | -1.22162 | 0.58266 |
| C | -0.13801 | 0.02478 | 1.17191 | C | -0.07856 | 0.07691 | 1.12421 | C | -0.09668 | 0.16026 | 1.21168 |
| C | -0.86372 | -2.07336 | -0.16908 | C | -0.93179 | -2.20027 | 0.13911 | C | -0.78896 | -2.32957 | 0.98115 |
| C | -0.51073 | -3.41331 | -0.0253 | C | -0.46339 | -3.50788 | 0.2699 | C | -0.08517 | -3.50549 | 1.23197 |
| N | 0.78291 | -3.52045 | 0.54186 | N | 0.90131 | -3.50236 | 0.64594 | N | 1.30372 | -3.29045 | 1.06436 |
| C | 1.19981 | -2.21278 | 1.02381 | C | 1.2694 | -2.15186 | 1.03452 | C | 1.55468 | -1.8611 | 0.96164 |
| C | -2.07019 | -1.73907 | -0.7553 | C | -2.23943 | -1.97813 | -0.24913 | C | -2.16955 | -2.33336 | 1.06217 |
| C | -2.91107 | -2.7702 | -1.16753 | C | -3.05718 | -3.08245 | -0.48006 | C | -2.81038 | -3.52268 | 1.40135 |
| C | -2.5394 | -4.10099 | -1.02348 | C | -2.56872 | -4.37697 | -0.35974 | C | -2.09134 | -4.6889 | 1.62866 |
| C | -1.31852 | -4.4479 | -0.45187 | C | -1.2494 | -4.61318 | 0.0163 | C | -0.70263 | -4.70024 | 1.54131 |
| C | 1.78139 | -4.14926 | -0.17749 | C | 1.85759 | -4.08523 | -0.16081 | C | 1.97759 | -4.00688 | 0.09463 |
| C | 3.04374 | -3.2898 | -0.11017 | C | 3.07225 | -3.15509 | -0.21965 | C | 2.89445 | -3.04973 | -0.66526 |
| N | 2.60496 | -2.15166 | 0.68638 | N | 2.63425 | -2.01947 | 0.58273 | N | 2.60961 | -1.77161 | -0.02476 |
| O | 1.7148 | 1.31275 | -1.6117 | O | 1.22803 | 1.03469 | -2.06879 | O | 0.05233 | 0.66958 | -2.31503 |
| O | -1.27179 | 1.276 | -1.36784 | O | -1.71959 | 0.99809 | -1.02522 | O | 0.98801 | 3.26464 | 1.44403 |
| O | 1.66863 | -5.19017 | -0.76547 | O | 1.75412 | -5.14542 | -0.71526 | O | 1.8562 | -5.18272 | -0.11635 |
| C | 4.18314 | -4.04969 | 0.5701 | C | 4.29824 | -3.83691 | 0.39009 | C | 4.35681 | -3.464 | -0.4942 |
| C | 3.40396 | -2.85114 | -1.53316 | C | 3.30411 | -2.73922 | -1.67481 | C | 2.46647 | -3.02494 | -2.13542 |
| C | 3.34298 | -1.09267 | 1.07401 | C | 3.35586 | -0.96073 | 1.00077 | C | 3.32113 | -0.63266 | -0.14419 |
| O | 2.88223 | -0.18488 | 1.73703 | O | 2.88241 | -0.11309 | 1.73278 | O | 3.07964 | 0.3551 | 0.51955 |
| C | 0.42579 | 3.38118 | 2.51446 | C | 1.33652 | 3.91704 | 1.25697 | C | -2.47033 | 1.32145 | -1.17139 |
| O | 0.60929 | 2.34172 | 3.11003 | O | 2.34967 | 3.26147 | 1.17587 | O | -2.4895 | 0.2643 | -0.57283 |
| N | 0.72798 | 4.59613 | 3.00972 | N | 1.25211 | 5.0521 | 1.98144 | N | -3.18592 | 1.57581 | -2.27532 |
| H | -2.72474 | 2.82319 | -2.84425 | H | -3.81483 | 2.20732 | -1.90295 | H | 0.7776 | 5.81706 | 1.69051 |
| H | -3.78277 | 5.00437 | -3.41211 | H | -5.3047 | 4.18871 | -2.12754 | H | -0.25313 | 8.02439 | 1.18208 |
| H | -3.4102 | 6.96887 | -1.96594 | H | -4.64836 | 6.34568 | -1.1244 | H | -2.19577 | 8.15794 | -0.33541 |
| H | -1.99879 | 6.77803 | 0.06677 | H | -2.49914 | 6.55524 | 0.09984 | H | -3.13764 | 6.09582 | -1.34618 |
| H | 1.45429 | 1.50876 | 1.1458 | H | 1.5253 | 1.48054 | 0.68839 | H | 1.26871 | 1.44126 | 0.1484 |
| H | 0.16874 | -0.10853 | 2.20954 | H | 0.37008 | 0.04263 | 2.11748 | H | 0.53949 | 0.34229 | 2.07768 |
| H | -1.21866 | 0.16779 | 1.13983 | H | -1.15361 | 0.22245 | 1.23026 | H | -1.14246 | 0.24233 | 1.51011 |
| H | 1.06871 | -2.13853 | 2.11238 | H | 1.21751 | -2.03038 | 2.15087 | H | 1.90462 | -1.45342 | 1.9203 |
| H | -2.34904 | -0.70411 | -0.9067 | H | -2.62043 | -0.97463 | -0.38534 | H | -2.74707 | -1.44254 | 0.85702 |
| H | -3.86463 | -2.52761 | -1.62233 | H | -4.08793 | -2.92538 | -0.77653 | H | -3.89168 | -3.53809 | 1.47591 |
| H | -3.20529 | -4.88346 | -1.3694 | H | -3.22117 | -5.21761 | -0.56719 | H | -2.61717 | -5.60479 | 1.87332 |
| H | -1.00171 | -5.47826 | -0.35856 | H | -0.84524 | -5.61403 | 0.09263 | H | -0.15006 | -5.60238 | 1.69346 |
| H | 5.10271 | -3.46627 | 0.54971 | H | 5.18235 | -3.20955 | 0.28431 | H | 5.01056 | -2.85366 | -1.11545 |
| H | 4.34555 | -4.98853 | 0.04166 | H | 4.46877 | -4.78103 | -0.12627 | H | 4.46109 | -4.50735 | -0.79005 |
| H | 3.92236 | -4.26525 | 1.6056 | H | 4.12823 | -4.03618 | 1.44752 | H | 4.65612 | -3.35941 | 0.54775 |
| H | 2.60022 | -2.23874 | -1.94018 | H | 2.4325 | -2.20179 | -2.04557 | H | 1.44166 | -2.66369 | -2.21131 |
| H | 4.32452 | -2.2692 | -1.53017 | H | 4.17674 | -2.09193 | -1.74912 | H | 3.11688 | -2.36438 | -2.70684 |
| H | 3.53661 | -3.73274 | -2.15887 | H | 3.46117 | -3.6295 | -2.28222 | H | 2.52371 | -4.03246 | -2.545 |

| | | | | | | | | | | | |
|---|---|---|---|---|---|---|---|---|---|---|---|
| H | 4.39608 | -1.1385 | 0.74241 | H | 4.39323 | -0.94113 | 0.62379 | H | 4.12498 | -0.68421 | -0.90006 |
| H | 0.39467 | 5.41583 | 2.53001 | H | 0.44994 | 5.64826 | 1.85933 | H | -3.14711 | 2.48544 | -2.69962 |
| H | 1.02894 | 4.65281 | 3.96556 | H | 2.10889 | 5.42777 | 2.34667 | H | -3.74452 | 0.84327 | -2.67196 |
